# Correcting batch effects in large-scale multiomics studies using a reference-material-based ratio method

**DOI:** 10.1186/s13059-023-03047-z

**Published:** 2023-09-07

**Authors:** Ying Yu, Naixin Zhang, Yuanbang Mai, Luyao Ren, Qiaochu Chen, Zehui Cao, Qingwang Chen, Yaqing Liu, Wanwan Hou, Jingcheng Yang, Huixiao Hong, Joshua Xu, Weida Tong, Lianhua Dong, Leming Shi, Xiang Fang, Yuanting Zheng

**Affiliations:** 1 State Key Laboratory of Genetic Engineering, School of Life Sciences and Human Phenome Institute, Shanghai Cancer Center, Fudan University, Shanghai, China; 2 Greater Bay Area Institute of Precision Medicine, Guangzhou, Guangdong China; 3 Division of Bioinformatics and Biostatistics, National Center for Toxicological Research, US Food and Drug Administration, Jefferson, AR USA; 4 National Institute of Metrology, Beijing, China; 5 International Human Phenome Institutes, Shanghai, China

**Keywords:** Batch effect, Ratio, Reference materials, Multiomics, Phenomics, Differentially expressed, Prediction, Data integration, Quartet family, Metrology

## Abstract

**Background:**

Batch effects are notoriously common technical variations in multiomics data and may result in misleading outcomes if uncorrected or over-corrected. A plethora of batch-effect correction algorithms are proposed to facilitate data integration. However, their respective advantages and limitations are not adequately assessed in terms of omics types, the performance metrics, and the application scenarios.

**Results:**

As part of the Quartet Project for quality control and data integration of multiomics profiling, we comprehensively assess the performance of seven batch effect correction algorithms based on different performance metrics of clinical relevance, i.e., the accuracy of identifying differentially expressed features, the robustness of predictive models, and the ability of accurately clustering cross-batch samples into their own donors. The ratio-based method, i.e., by scaling absolute feature values of study samples relative to those of concurrently profiled reference material(s), is found to be much more effective and broadly applicable than others, especially when batch effects are completely confounded with biological factors of study interests. We further provide practical guidelines for implementing the ratio based approach in increasingly large-scale multiomics studies.

**Conclusions:**

Multiomics measurements are prone to batch effects, which can be effectively corrected using ratio-based scaling of the multiomics data. Our study lays the foundation for eliminating batch effects at a ratio scale.

**Supplementary Information:**

The online version contains supplementary material available at 10.1186/s13059-023-03047-z.

## Background

Batch effects are notorious technical variations irrelevant to study factors of interests, but are common in transcriptomics [1–5], proteomics [6–9], metabolomics [10–12], and multiomics integration [13]. Due to variation in experimental design, lab conditions, reagent lots, operators, and other non-biological factors, results from different batches may vary and result in misleading outcomes [11, 14–18].

Batch effects can have a profound negative impact on study outcomes [16, 17]. On the one hand, the presence of batch-correlated variations can skew analysis and introduce large numbers of false-positive or false-negative findings, and even mislead conclusions [19]. For example, a change of experimental solution caused a shift in the calculated patient risk, leading to incorrect treatment decision [20]. On the other hand, systematic variations including batch effects have become one of the major causes of irreproducibility [21, 22]. What is worse, reproducibitily crisis raises questions about the reliability of omics data and whether data collected from different batches or platforms are comparable for the intended research purpose. For example, researchers often opt to profile the same samples with RNAseq that were previously profiled with microarrays in order to avoid batch effects introduced by the inherent differences between the two technology platforms for transcriptomic analysis. Such a costly undertaking may be averted when data from distinct platforms can be integrated properly [23, 24]. With the era of big data flooded with multiomics data, the issue of batch effects becomes more prominent [16, 25].

Although many batch-effect correction algorithms (BECAs) have been proposed [11, 15, 26–29], studies that aim to comprehensively assess the performance of various BECAs for applications to multiomics studies are currently lacking, or have yielded controversial results. For example, in transcriptomics, several widely used BECAs, such as ComBat [27, 30], surrogate variable analysis (SVA) [29], and RUVseq [28], have been shown acceptable performance in some studies [31–33], but did not perform well in others [16, 34, 35]. Similarly, the ratio-based method by scaling feature values relative to those of common reference sample(s), which is also known as Ratio-G, has shown improved comparability in some multi-batch studies [1, 4, 36], but not in other studies [34]. Recently, Harmony, a method based on dimensionality reduction by principal component analysis (PCA), has shown to perform well in batch-group balanced and confounded scenarios in single-cell RNAseq data [37, 38]. However, it remains to be seen whether Harmony works well for other omics data types.

The nature of the datasets used for performance comparison of the BECAs in previous studies is insufficient to determine the actual cause of batch effects. For example, several studies were based on different biological samples [38, 39], which were difficult to assess pure batch variations against hidden subpopulation variabilities among batches. And some studies were based on simulated datasets [34, 39], which do not necessarily accurately represent the true nature of batch effects. These datasets used in the previous studies could not objectively reflect the nature of batch effects and might lead to biases in performance evaluation of the BECAs. Therefore, studies based on real-world, cross-batch datasets are urgently needed for objective performance assessment of the BECAs.

Moreover, the levels of confounding between biological and batch factors may greatly influence the validity of BECAs. In a balanced scenario where samples across biological groups of interest are evenly distributed across batch factors, batch effects can be mitigated via diverse BECAs [16, 34, 40]. The balanced scenario is ideal but almost impossible in reality. In most cases, biological factors and batch factors are often mixed and difficult to distinguish, which is recognized as the confounded scenario and is commonly seen in longitudinal and multi-center cohort studies. When biological factors and batch factors are strongly confounded, most BECAs may no longer be applicable [16, 34]. Therefore, there is an urgent need to identify batch correction methods to facilitate the integration of datasets from confounded batch-group scenarios.

Here, as part of the Quartet Project for quality control and data integration of multiomics profiling [41], we comprehensively assessed the performance of seven BECAs for mitigating the impact of batch effects in multiomics datasets, including transcriptomics, proteomics, and metabolomics data. We previously established and well characterized the first suites of publicly available multiomics reference materials of matched DNA [42], RNA [43], protein [44] and metabolite [45] derived from the same B-lymphoblastoid cell lines from the four members of a monozygotic twin family [41]. A large number of multiomics datasets were generated from multiple labs, platforms, and protocols. These rich datasets provided a unique opportunity for us to objectively assess the performance of BECAs based on the underlying nature of batch effects under both balanced and confounded scenarios. The performance was evaluated in terms of the reliability of identifying differentially expressed features (DEFs), the robustness of predictive models, and the classification accuracy after multiomics data integration. Our findings show the promise for eliminating batch effects and enhancing data integration in increasingly large-scale, cross-batch multiomics studies.

## Results

### Overview of the study design

**Fig. 1:**
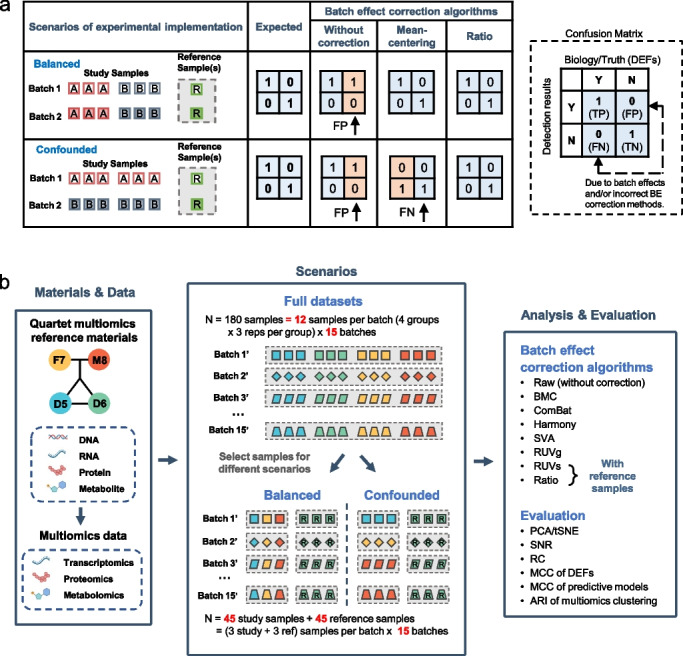
Overview of the study design. **a** Advantages and limitations of batch-effect correction algorithms (BECAs) under balanced and confounded experimental scenarios. False positives and false negatives in cross-batch comparisons using different BECAs. **b** Overview of datasets and analysis approaches. Multi-batch datasets from transcriptomics, proteomics, and metabolomics were generated using the Quartet multiomics reference materials derived from a Quartet family including father (F7), mother (M8), and monozygotic twin daughters (D5 and D6). Subsets of data were selected from the full datasets to create balanced and confounded scenarios for assessing the pros and cons of BECAs. The multiomics profiling data were analyzed with seven BECA methods. Performances were then evaluated using visualization tools and quantitative metrics

Advantages and limitations of BECAs under balanced and confounded scenarios were shown in Fig. 1a. Suppose we have a total of 12 samples from two groups (A and B), including six As and six Bs from two batches, and the objective is to detect DEFs between group A and group B. Ideally, in a balanced scenario where the two batches contain an equal number of replicates from both groups A and B, batch effects can be effectively corrected by many batch-effect removal methods, such as mean-centering per feature per batch. However, experimental scenarios are rarely balanced. In an extreme scenario when the sample group is completely confounded with the batch number in that all six As are processed in one batch and all six Bs in another batch. Then, it is almost impossible to distinguish the real biological differences between A and B from technical variations resulting from batch effects. In this case, an incorrect combination of scenario-methods can lead to false negatives, because the true biological differences between the two groups can be removed during the removal of batch effects. An effective way of tackling batch effects is to concurrently profile one or more reference material(s) (*e.g.*, one chosen Quartet multiomics reference material) along with the study samples in each batch. Expression profiles of each sample can be transformed to ratio-based values using expression data of the reference sample(s) as the denominator, whether in balanced or confounded scenarios (Fig. 1a).

To objectively assess performance of the BECAs, multiomics and multi-batch datasets based on the Quartet reference materials were used (Fig. 1b). As described in accompanying papers [41–46], complete suites of DNA, RNA, protein, and metabolite reference materials were established simultaneously from four immortalized B-lymphoblastoid cell lines (LCLs) derived from a Quartet family including monozygotic twin daughters (D5 and D6) and their father (F7) and mother (M8). Reference materials were then distributed to multiple labs for generating multiomics data. For each omics type, 12 libraries from 12 vials with each representing one of the triplicates of a donor were used for concurrent data generation in a batch. On the other hand, high-throughput experiments at different time points, in different labs, using different platforms or experimental protocols were recognized broadly as cross-batch experiments. Finally, multiomics datasets, including transcriptomics, proteomics, and metabolomics datasets from multiple labs, platforms, protocols, and batches were obtained, comprising a total of 252 RNA libraries from 21 batches [43], 384 protein libraries from 32 batches [41, 44], and 264 metabolite libraries from 22 batches [45]. For each omics type, 15 batches of data from different platforms, labs and with different data quality were employed and referred as full datasets for further analysis (Fig. 1b and Additional file 1: Fig. S1).

We then employed a subset of datasets from the full datasets to create balanced and confounded scenarios for assessing the pros and cons of the BECAs (Fig. 1b). Here, we arbitrarily used D6 as the common reference material, leaving the rest three as the study groups (D5, F7, and M8). In the balanced experiment scenario, one replicate was selected for each study group from each of 15 batches. This was done independently for each omics type. In the confounded experiment scenario, five batches were randomly assigned to each study group (D5, F7, or M8) for each omics type to extract all three replicates for the assigned study group. For both scenarios, all three replicates for the selected reference sample (D6) in each batch were retained for reference-sample-based BECAs. Therefore, 45 study samples and 45 reference samples in balanced and confounded scenarios were employed at each omics level (Fig. 1b).

We evaluated seven BECAs, including per batch mean-centering (BMC), ComBat [30], Harmony [47], SVA [29], RUVg [28], RUVs [28], and ratio-based scaling (see Methods for details). We visualized clustering projections with both PCA and t-distributed stochastic neighbor embedding (t-SNE). We also applied five quantitative metrics for performance evaluation. First, signal-to-noise ratio (SNR) was used for quantifying the ability to separate distinct biological groups when multiple batches of data were integrated. Secondly, the relative correlation (RC) coefficient between a dataset and the reference datasets in terms of fold changes (FC) was used to measure their consistency. Thirdly, Matthews correlation coefficient (MCC) was used to measure the consistency between a dataset and the reference dataset in terms of DEFs as the truth. The reference datasets were generated from the consensus of DEFs from intra-batch profiling from the full datasets. Fourth, MCC was used to represent the predictivity of models for predicting the sex and age of the donors from whom the reference materials were derived. Finally, adjusted Rand index (ARI) was used for measuring the accuracy of classification after multiomics data integration (Fig. 1b).

### Multiomics measurements are prone to batch effects and can be corrected using appropriate methods

**Fig. 2:**
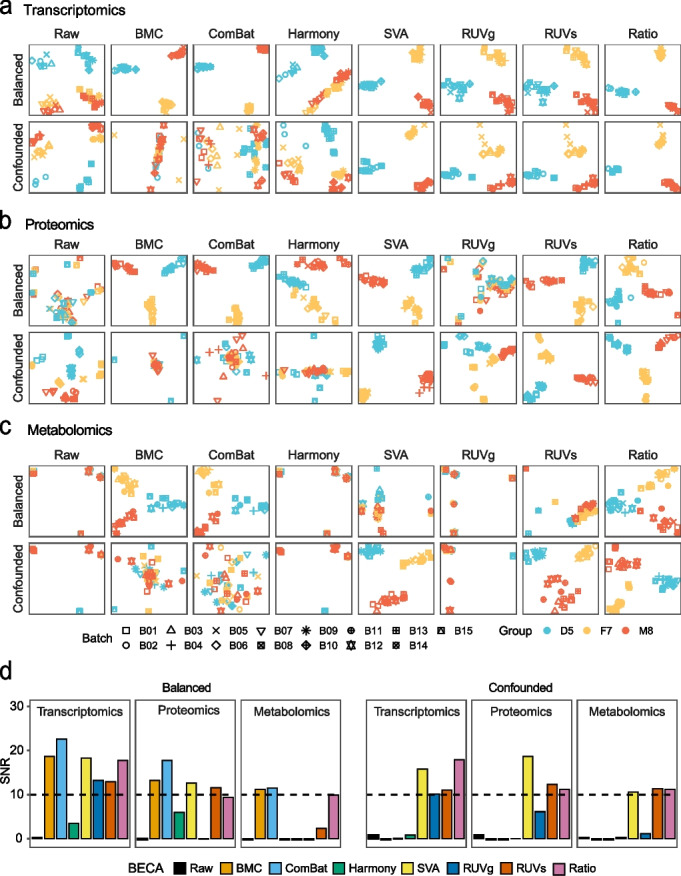
Multiomics measurements are prone to batch effects and can be corrected using appropriate methods. **a**-**c** PCA plots based on different batch-effect correction algorithms (BECAs) in balanced and confounded scenarios, using transcriptomics (**a**), proteomics (**b**), and metabolomics (**c**) data. Plots were color-coded by donor (D5, F7, and M8), and shaped by batch. (**d**) Bar plot of signal-to-noise ratio (SNR) using different BECAs on transcriptomics, proteomics, and metabolomics data

We first applied PCA scatter plots to visualize the magnitude of biological and (or) batch effects (Fig. 2a-c). In transcriptomics, it could be observed that, without correction, experimental factors rather than biological groups (D5, F7, or M8), exhibited the largest differences. BMC and ComBat performed well in distinguishing libraries according to their biological groups only in the balanced scenario (Fig. 2a top), not in the confounded scenario (Fig. 2a bottom). In contrast, the other four BECAs, including two BECAs with reference samples (RUVs and ratio-based scaling), RUVg, and SVA performed equally well in both balanced and confounded scenarios (Fig. 2a and Additional file 1: Fig. S2a). Similar results were observed in proteomics (Fig. 2b and Additional file 1: Fig. S2b). In metabolomics, Harmony, SVA, RUVg, and RUVs did not perform as well as in transcriptomics, probably because they were developed primarily with transcriptomic data (Fig. 2c and Additional file 1: Fig. S2c) and the level of batch effects in metabolomic data is generally higher (Fig. 2c left). The performance of the straightforward methods such as BMC and ratio-based scaling was omics-independent, *i.e.*, similar trend of performance was observed in transcriptomics, proteomics, and metabolomics.

The performance metric of SNR based on PCA was then used to quantify differences between biological sample groups and variations in technical replicates. SNR measures the ability of distinguishing intrinsic biological differences among distinct donors (“signal”) from technical variations including batch effects of the same donor (“noise”), as mentioned in the accompanying papers [41, 43]. Generally, a higher SNR value indicates higher distinguishing power, and vice versa. SNR values were consistently high in ratio-based scaling in balanced and confounded scenarios among the three omics types, whereas SNR values of SVA, RUVg, and RUVs were high for only one or two omics types, but low for the others. On the other hand, SNR values of BMC and ComBat were high for balanced scenario but consistently low in confounded scenario in all three omics types (Fig. 2d).

### Reliability of identifying differentially expressed features

**Fig. 3:**
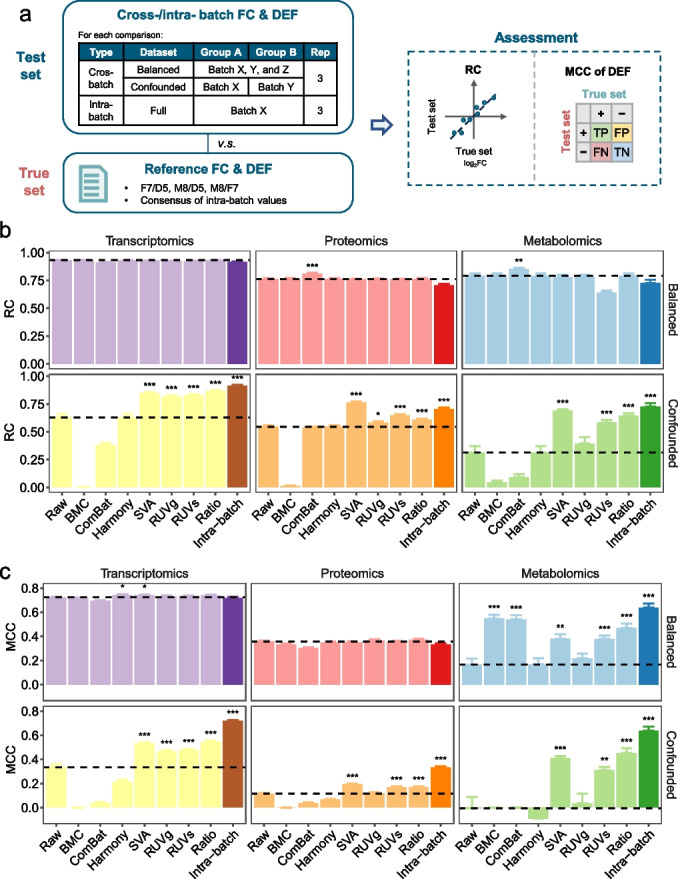
Reliability of identifying differentially expressed features. **a** Schematic diagram of comparisons of differentially expressed features (DEFs) between cross-batch and intra-batch datasets with reference datasets. **b**-**c** Bar plots (mean ± s.e.) representing the relative correlation (RC) (**b**) and Matthews Correlation Coefficient (MCC) of DEFs (**c**) with reference datasets and across seven BECAs in balanced and confounded scenarios using transcriptomics, proteomics, and metabolomics data. Mean value of the dataset without correction (raw) in each panel was plotted in dashed line. Performances between the raw group and BECA groups were compared using Student’s t-test. A group with the performance significantly higher than raw group was marked with stars (*). Symbolic number coding of *p*-value was used as: *** (*p* ≤ 0.001), ** (0.001 < *p* ≤ 0.01), * (0.01 < *p* ≤ 0.05)

As identifying DEFs is one of the most important tasks for quantitative omics, we compared performance in DEF identification across batches, using the reference FCs and DEFs as the “ground truth” for benchmarking (Fig. 3a, see Methods for details). Reference FCs and DEFs of three donor-pairs (F7/D5, M8/D5, and M8/F7) were constructed using a consensus-based strategy (Additional file 1: Fig. S3).

We then developed two quality metrics, namely relative correlation (RC) and MCC of DEFs. Specifically, we introduced the RC metric, *i.e.*, the Pearson correlation coefficient between the FCs of a test dataset for a given pair of donors and the corresponding reference FCs. In addition, we used the “MCC of DEFs” metric, *i.e.*, MCC to measure the consistency of DEFs detected from a test dataset for a given pair of donors with those from the reference DEFs (Fig. 3a). Intra-batch RC and MCC of DEFs which was calculated based on the full datasets comprising 12 libraries (4 donors × 3 replicates) in each batch were used as the positive control.

In balanced scenario, RC values were equally good with or without BECAs. Most of the BECAs showed no significant difference in performance, except for ComBat which showed higher performances in proteomics and metabolomics (Fig. 3b). On the contrary, SVA, RUVs, and ratio-based scaling exhibited significantly higher RC values in confounded scenario across the three omics types (*p* < 0.001) (Fig. 3b). Moreover, some BECAs were able to improve RC values as high as those in intra-batch, while others significantly reduced RC values, highlighting the importance of choosing a suitable BECA in order to avoid negative impact, especially in confounded scenario (Fig. 3b). Using MCC of DEFs as a metric, SVA, RUVs and ratio-based scaling consistently outperformed other methods in confounded scenario, which was in line with the assessment by RC (Fig. 3c). Indeed, when applying a variety of widely used performance metrics, such as sensitivity, specificity, precision, and Jaccard Index of DEFs, we observed similar performance across different BECA methods (Additional file 1: Fig. S4). Moreover, we applied false-positive rate (FDR) and nonstringent *p* value for statistical analysis in identifying DEFs to compare the performances between with or without controlling multi-testing. Using MCC of DEFs as a metric, the performances across BECAs remained unchanged whether with or without controlling for multi-testing (Additional file 1: Fig. S5).

### Reliability of model prediction

Cross-batch prediction, *i.e.*, developing a prediction model based on data from one batch and validating its performance based on data from another batch, is another important task in quantitative omics, especially in the context of biomarker discovery for clinical diagnosis, prognosis, and therapeutic action. Thus, we evaluated the impact of BECAs on cross-batch prediction performance.

Frequently, a predictive model was built using some dataset(s), and was further validated using independent dataset(s) [3, 4]. These datasets could be confounded with batch effects. In this study, we divided our dataset into two sets before developing predictive models. Specifically, 27 libraries from nine batches were used as the training set and 18 libraries from six batches as the validation set, according to data generation date, as we did in MAQC-II [3]. The training set was used to train prediction models using five machine-learning algorithms, including model averaged neural network (avNNet), support vector machine (SVM), random forest (RF), generalized partial-least squares (GPLS), and linear algorithm BstLm, through an internal-layer of 25 runs of fivefold cross-validation process to resist overfitting. A model was further validated using the validation set as an external-layer of evaluation. Age and sex of the donors from whom the Quartet reference materials were developed were used as the biological endpoints to assess the robustness of cross-batch prediction. Because sex-specific genes and age-related genes have been known, leading to these two endpoints (sex and age) are easy to predict compared to most clinically relevant endpoints such as disease subtyping. Thus, a failure of accurate prediction of these easy endpoints would imply serious problems in clinical settings.

**Fig. 4:**
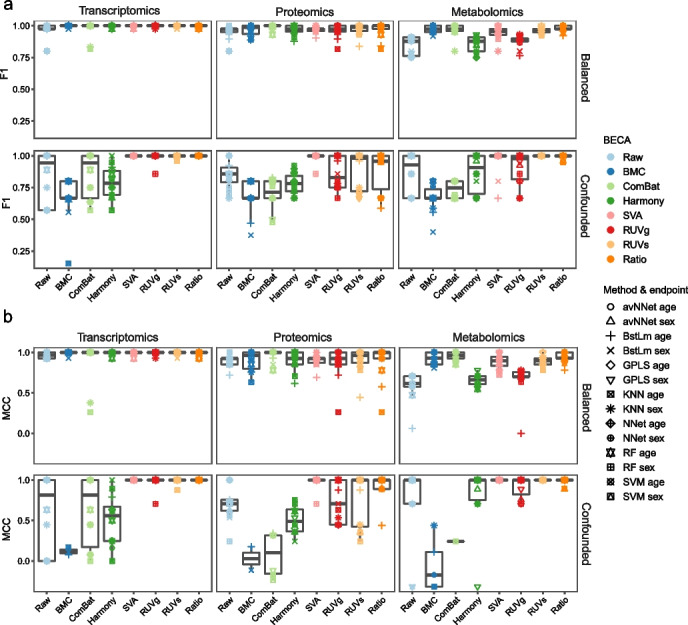
Reliability of model prediction. Validation performances, measured using F1 scores (**a**) and MCC (**b**), in predicting sex and age under balanced and confounded scenarios. According to data generation date, 27 libraries from the former nine batches were used as training set, while the remaining 18 libraries from the latter six batches were used as validation set. Prediction models were constructed based on training set using five machine-learning algorithms, including model averaged neural network (avNNet), support vector machine (SVM), random forest (RF), generalized partial least squares (GPLS), and linear algorithm BstLm. The models were then validated using the validation set and calculated the performances

Based on multiple evaluation metrics, we found that under the balanced scenario, the prediction performance of a machine learning method was equally good with or without BECAs, and there were no differences in performance among the BECAs (Fig. 4 and Additional file 1: Fig. S6). On the other hand, under the confounded scenario, SVA, RUVs, and ratio-based scaling performed well, whereas BMC, ComBat, and Harmony performed as bad as or even worse than non-correction (Fig. 4 and Additional file 1: Fig. S6). This trend remained consistent for transcriptomics, proteomics, and metabolomics.

### Consistency of multiomics clustering

**Fig. 5:**
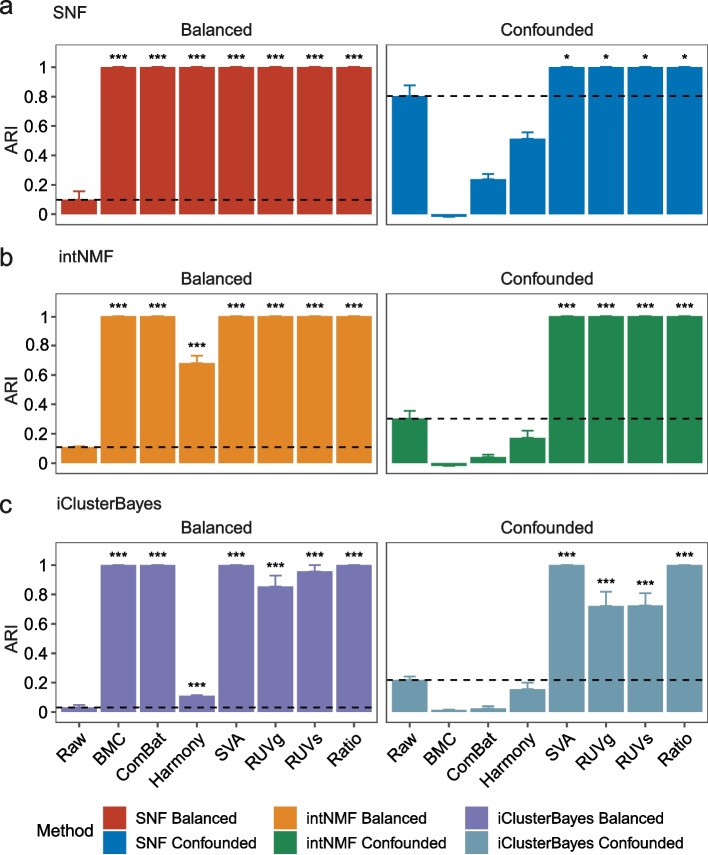
Consistency of multiomics clustering according to their donors. Bar plots (mean ± s.e.) of Adjusted Rand Index (ARI) values of multiomics clustering using different batch-effect correction algorithms in balanced and confounded scenarios. Three integrative tools were used, including SNF (**a**), intNMF (**b**), and iClusterBayes (**c**). Expression profiles from 36 samples from three donors derived from 12 batches in each omics type were randomly selected from the balanced and confounded datasets and further used to integrate cross-omics data. In order to eliminate selection bias, the random selection and cross-omics integration was conducted ten times. Mean value of the dataset without correction (raw) in each panel was plotted in dashed line. Performances between the raw group and BECA groups were compared using Student’s t-test. A group with the performance significantly higher than the raw group was marked with stars (*). Symbolic number coding of *p*-value was used as: *** (*p* ≤ 0.001), ** (0.001 < *p* ≤ 0.01), * (0.01 < *p* ≤ 0.05)

As clustering multiomics data has the potential to identify disease subtypes and to reveal systems level insights, it has become one of the most popular applications in integrative analysis. Hence, we further compared performance of these BECAs in terms of ability to accurately clustering cross-batch libraries into their donors (D5, F7, and M8) after multiomics data integration. Datasets consisting 36 libraries of three donors derived from 12 batches were randomly selected from the entire dataset in balanced and confounded scenarios (see Methods for details). Three widely-used integrative tools were used, including SNF [48] (Fig. 5a), intNMF [49] (Fig. 5b), and iClusterBayes [50] (Fig. 5c). The true labeling was set to three donors, with one group corresponding to the multiomics samples of the same group, because the Quartet multiomics materials, including RNA, protein and metabolite, were derived from the same batch of cultured cells and were established in the same batch. Hence, it was expected that the same within-group similarity was maintained across omics layers. Replicates from the same donor should be clustered together, regardless of within-omics or cross-omics. The performance was measured using the ARI [51], a commonly used metric to compare the clustering labeling against the true labeling.

SVA and ratio-based scaling consistently performed equally well or better than other BECAs across the three integrative tools (Figs. 5a-c). BMC and ComBat showed excellent performance (ARI = 1) in the balanced scenario; however, they performed poorly (ARI around zero) in the confounded scenario. Additionally, RUVg and RUVs performed well using SNF and intNMF methods for multiomics integration (Figs. 5a-b), but were less effective for iClusterBayes method (Fig. 5c). The choice of different integrative tools showed modest differences to the results except for datasets after Harmony correction. Our results highlighted the problems of widely used BECAs in real-world scenarios where batch effects are prevalent.

### Overall performances of the BECAs

**Fig. 6:**
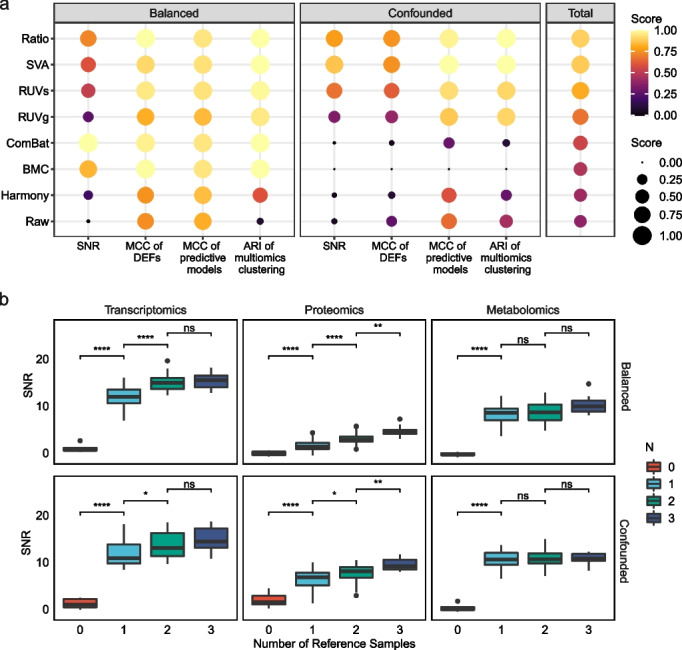
Summary of performances of BECAs and choice of number of samples for ratio-based scaling. **a** The summarized performance of seven BECAs in balanced and confounded scenarios. The BECAs were ordered by their total score. For the calculation of the total score, we first separately scaled the values of the four metrics, including signal-to-noise ratio (SNR), Matthews Correlation Coefficient (MCC) of identification of differentially expressed features (DEFs), MCC of prediction, and Adjusted Rand Index (ARI) of multiomics clustering, to an interval of (0,1) to equalize the weight of different metrics. The total score was expressed as mean of the scaled values of the four metrics. **b** Boxplot of SNR under different numbers of replicates of the reference sample used as dominators in conducting ratio-based scaling. Student's t-test was conducted. Symbolic number coding of *p*-value was used as: *** (*p* ≤ 0.001), ** (0.001 < *p* ≤ 0.01), * (0.01 < *p* ≤ 0.05), ns (*p* > 0.05, not significant)

We provided a summary of the overall performance of seven BECAs as measured by SNR, MCC for DEFs, MCC for model prediction, and ARI for multiomics data integration (Fig. 6a). Ratio-based scaling ranked on the top and exhibited a general superiority by significant improvements in SNR, identification of DEFs, cross-batch prediction and multiomics clustering, compared to raw data without correction. Besides, SVA, RUVs, and RUVg were alternative methods that were suitable in both balanced and confounded scenarios. ComBat and BMC were highly context-dependent and were only suitable in the balanced scenario. Harmony, a BECA method developed based on single-cell RNAseq data, showed limited improvement for bulk RNAseq, proteomics, and metabolomics data.

### Performance of ratio-based scaling with different number or quality of reference samples

If one uses reference materials to conduct ratio-based scaling, an important question is what number of replicates would constitute an appropriate choice as the denominator for converting absolute expression data to ratio-based scales. Thus, the number of reference samples that could be used as the denominator in ratio-based scaling within each batch was tested. As expected, SNR increased when using ratio-based expressions compared to absolute expressions even when only one replicate was used, and further increased when more replicates were added to calculate the average expression values as the denominator (Fig. 6b). These findings emphasized that it is critical to use reference samples per-batch along with study samples, and that it is better to use more reference samples and/or replicates to reach stability of the denominator.

The performance of the ratio-based method might also be affected by the quality of the reference sample. Here, we used two strategies for simulation of low-quality reference samples to evaluate the consistency of ratio-based approach under different data quality scenarios (see Methods for details). First, we artificially introduced different levels of noise to the reference samples to simulate low-quality samples caused by a lower measurement precision. Using SNR across study samples to demonstrate effectiveness of the ratio-based method, the results showed that the SNR value continuously decreased with deteriorating quality of reference samples (Additional file 1: Fig. S7). Moreover, with transcriptomics data as an example, when the noise level was increased up to threefold standard deviation (SD), the SNR values remained as high as 4.7 in balanced scenario and 10.3 in confounded scenario, which were much higher than original dataset without correction (SNR: -0.3 to -0.9). The results indicated that the ratio-based method performed reasonably well as long as the quality of reference samples was not extremely low. Additionally, SNR values based on noises with onefold SD were similar with real datasets, as expected, except in the balanced proteomics dataset. This might be explained by the fact that there were more low-quality datasets in proteomics compared to transcriptomics and metabolomics (Additional file 1: Figs. S1 a-c), and lower correlations of replicates of reference samples were detected (Additional file 1: Fig. S1d).

Secondly, we artificially reduced the expression level of some genes to simulate reference samples with low-quality in transcriptomics data. In transcriptomics, a low-quality sample is usually characterized with RNA degradation and such degradation is usually gene specific, that is, some genes are degraded more severely while others do not. The fragmentation of RNA molecules in the low-quality sample can result in a decrease in the complexity of the RNA library, leading to some RNA fragments not detectable and therefore lower count values in the RNAseq analysis [52, 53]. Here, for each replicate of the reference sample, we randomly selected a certain percentage of genes and artificially reduced their read counts to 1/2, 1/4, or 1/8 of the original levels (Additional file 1: Fig. S8). Similarly, SNR values was decreased when larger percentage of genes were reduced. However, it should be noticed that when up to 10% of detected genes were reduced to 1/8 of their original counts, the SNR values remained as high as 17.1 and 14.8 in balanced and confounded scenario, respectively, which were much higher than the original dataset without correction. Moreover, even when the percentage of affected genes were increased up to 30%, the SNR values in confounded scenario remained 14.5. The results indicated that as long as the quality of reference samples was not too low, the ratio-based method remained effective.

## Discussion

Batch effects in multiomics profiling are universal and detrimental to study purpose. Our results showed that batch effects were prevalent in quantitative profiling technologies, presenting challenges for combining data from different batches of single-omics and multiomics. Hence, batch correction is an essential step in multi-batch analysis.

Applying BECAs is highly context-dependent. In a balanced scenario, the batch effects are evenly distributed across study groups and can be got rid of via all seven BECAs we tested. In reality, however, the ideal batch-group design is almost impossible in multi-center and longitudinal cohort studies, when batch effects can be fully confounded with the investigational endpoints of interests. Furthermore, batch effects hamper the legitimacy of retrospective data integration aiming to explore new insights from comparison of several independent cohort studies, such as the healthy and disease cohorts [16]. In these cases, some BECAs, such as BMC and ComBat, were no longer applicable. What is worse, incorrect usage of BECAs could lead to many detrimental effects such as removal of true biological signals (Figs. 3, 4 and 5).

Our results indicated that the application of the ratio-based method is warranted. The ratio-based method scales the absolute feature values of study samples relative to those of concurrently measured reference sample(s) on a feature-by-feature basis and can effectively mitigate the widespread problems of batch effects, in transcriptomics, proteomics, and metabolomics datasets, especially in cases when batch effects are highly confounded with biological factors of interests.

Moreover, the effectiveness and broad applicability of the ratio-based method can be explained, at least partially, by the fundamental principles and assumptions behind data representation of omics measurements, as was described in Zheng et al. [41]. Briefly, a measured value of a gene (*I*) can be expressed as *I* = *f*(*C*), where *C* is the concentration or abundance of an analyte and *f* is assumed to be a linear and fixed relationship between *I* and *C* under any experimental conditions [54]. It should be noted that *f* is fixed or comparable under the same experiment (batch). However,* f* can vary across batches, due to large variations in experimental design, lab conditions, reagent lots, operators, and other non-biological factors. Hence, the measured value of a gene in batch 1 (*I*_1_) and batch 2 (*I*_2_) may be largely different and less reproducible. On the contrary, when a common reference sample (*r*) is profiled along with study samples in the same experiment (batch), ratio-based scaling can be applied and *f* becomes irrelevant for the ratio data. Thus, the resulting ratio of *I*^*s*^/*I*^*r*^ from each batch will remain reproducible and accurately reflect the ratio of *C*^*s*^/*C*^*r*^.

We prefer the ratio-based method for three reasons. First, the ratio-based method is easy to implement, platform-independent, and applicable to multiomics quantification, including transcriptomics, proteomics, and metabolomics. Secondly, compared to ComBat or BMC, the ratio-based scaling is less affected by study design of unbalanced distributions of samples in different sample groups between different batches. In clinical applications and large-scale projects, the imbalance of samples across different batches is inevitable. Thirdly, SVA is designed to remove all unwanted sources of variation and retain differences between the specified primary variables (biological) [29]. This may lead to removing potentially important biological information encoded in the latent variables. Thus, SVA is not appropriate for studies with unknown subgroups of biological interests [29], such as in molecular subtyping studies. In contrast, the ratio-based method removes batch effects by taking advantage of the fundamental characteristics of quantitative omics measurements using common reference sample(s), which means that it is hypothesis free and the important potential biological variables do not have to be known in advance.

According to our results, using two or three replicates of common reference material(s) in each batch and converting expression data to feature-wise ratio-based scaling profiles within each batch can play an important role in making expression levels inherently more comparable and hence resistant to batch effects. As the Quartet multiomics reference materials and the corresponding reference datasets have been successfully developed in our accompanying work [41–45], which represent the first suites of publicly available multiomics reference materials, we therefore recommend the use of the Quartet reference materials or equivalents for monitoring and correcting batch effects. Furthermore, the DNA and RNA reference material suites have been certified by China’s State Administration for Market Regulation as the First Class of National Reference Materials and are extensively being utilized for proficiency testing and method validation. Profiling Quartet reference materials along with study samples in each batch can be used not only for monitoring and correcting batch effects, but also proficiency testing and internal quality control.

Based on the experimental design composed of specific batches of samples representing balanced or confounded distributions of biological groups, we used standard analysis types such as sample clustering, differential analysis and predictive modelling to demonstrate comprehensive evaluations of some widely-used BECAs. Several findings in this study are consistent while some are controversial with previous reports. For example, Luo et al*.* [4] focused on performances of cross-batch prediction in clinical outcomes, and found that ratio-based method outperformed others. Moreover, Weishaup et al*.* [31] demonstrated the effectiveness of the RUV method in removing batch effect for molecular subtyping based on various microarray datasets of cerebellar and medulloblastoma, compared to without batch correction, which is consistent with our findings. On the other hand, Zhou et al. [34] simulated balanced and confounded datasets and found that BECAs performed well when batch-class was balanced, which is in line with our findings. However, when batch-class was strongly confounded, they found the performances declined in all BECAs under tested, including ComBat, SVA and ratio-based method, which appeared to be inconsistent with our findings. This might have been caused by the assumptions underlying the simulated datasets used for evaluation.

While the ratio-based method performed favorably in both balanced and confounded scenarios, it is not free of limitations. First, some features cannot be successfully corrected, for example, features that are not expressed in the reference material, including cell-specific features in numerator or male-specific feature when using a female material as the dominator. However, ratio-based scaling can successfully mitigate batch effect from numerous features when expressed in both the study samples (numerator) and reference material(s). Secondly, a reference-based method is possible when the introduction of a reference sample can be decided as part of the experimental design. It is not applicable when combining already existing dataset as the reference sample may not exist or be possible. Thirdly, applying ratio-based scaling needs to include reference samples in each batch, which means additional cost even if limited. Take RNAseq as an example, users can apply two or three samples/replicates per batch of 96 libraries for ratio-based expression profiling, resulting in a reasonable additional cost of 3.2% (3/(96–3)). Additionally, when well-established reference materials are used, such as Quartet multiomics reference materials, users can monitor batch quality based on pre-defined ‘ground truth’ in reference materials for proficiency testing and quality control, which can take full use of reference samples. Fourthly, the performance of the ratio-based scaling can be affected by the quality of the reference sample. If the quality of the reference sample is not good, the power of ratio-based method will be compromised. One possible solution is to use multiple (for example, two or three) replicates of reference samples and take the averaged value as the denominator. Finally, the ratio method assumes that there is no interaction between batch and the reference sample, that is, the reference sample is invariant across batches or times. Here, considering the concurrent availability of large amounts of the Quartet multiomics reference materials with demonstrated short- and long-term stability, we therefore recommend using the Quartet multiomics reference materials for monitoring and correcting batch effects. The limitations and caveats of our reference materials and the ratio approach warrant further investigations.

Furthermore, our study bears some limitations. First, the number of samples used for developing predictive models was small and the biological endpoints (sex and age) were relatively easy to predict. For example, the MAQC/SEQC Consortia comprehensively assessed the prediction performances of classification models from multiple analysis teams based on microarray and RNAseq datasets, and found that sex was the easiest to predict (MCC: 0.839 ~ 0.973) compared to clinically relevant endpoints (MCC: 0.129 ~ 0.748) [3, 23]. Additionally, the accuracy of predictive models for distinguishing between young and old individuals was high (AUC: 0.95) [55]. Hence, the performance presented here could be considered as an upper bound of the respective methods being investigated, which could not fully represent clinical applications. Secondly, samples used in the study were derived from the Quartet reference materials. Although clear trend of pros and cons across BECAs could be observed, a larger sample size and more tissue types of samples should be included in further investigations.

## Conclusions

In summary, multiomics measurements are prone to batch effects, which, fortunately, can be effectively corrected by using ratio-based scaling of the multiomics data. Profiling common reference materials concurrently with study samples can enhance data comparability of multi-batch studies, especially for large-scale multiomics studies, helping the discovery and validation of omics-based biomarkers for precision medicine.

## Methods

### Quartet reference materials

Quartet multiomics reference materials were derived from the same batch of immortalized Epstein-Barr Virus (EBV) infected B-lymphoblastoid cell lines (LCLs) from a four-member Chinese Quartet family, including two monozygotic twin daughters (D5 and D6), their father (F7), and their mother (M8). Cell line authentication was conducted and described by Zheng et al. [41]. Briefly, based on profiling of 15 short tandem repeat (STR) loci, we found that there were no differences between DNAs isolated from LCLs and their primary blood samples. Moreover, results based on STR analysis confirmed the relationship between the four Quartet samples, that was, F7 and M8 were biological parents of D5 and D6, while D5 and D6 were identical twins.

Multiomics reference materials, including DNA, RNA, protein, and metabolite, were established from the same batch of cultured cells. Large amounts of the reference materials with demonstrated short- and long-term stability were obtained, providing material basis for the batch monitoring and correction. More information of reference materials was detailed in the accompanying papers of Quartet Project, including the overall study [41], DNA [42], RNA [43], protein [44] and metabolite [45].

Importantly, Quartet DNA and RNA reference materials have been certified by China’s State Administration for Market Regulation as the First Class of National Reference Materials and are extensively being utilized for proficiency testing and method validation. The certified reference material numbers are GBW09900 (DNA of F7), GBW09901 (DNA of M8), GBW09902 (DNA of D5), GBW09903 (DNA of D6), GBW09904 (RNA of F7), GBW09905 (RNA of M8), GBW09906 (RNA of D5), and GBW09907 (RNA of D6).

Reference materials were then distributed to multiple labs for generating multiomics profiling data. According to the Quartet Project study design, in each omics type, 12 samples were used as a standard sample set, consisting of 12 tubes with each representing one of the triplicates of a donor [41]. The high-throughput experiments were conducted concurrently for the 12 samples. On the other hand, high-throughput experiments at different time points, in different labs, using different platforms or experimental protocols are recognized broadly as cross-batch experiments.

Finally, a large quantities of multiomics datasets were obtained, comprising of 252 RNAseq profiles from 21 batches [43], 384 LC–MS/MS proteomics profiling from 32 batches [41, 44], and 264 LC–MS/MS based metabolomics profiling from 22 batches [45]. The high-throughput datasets are deposited in the Quartet Data Portal (http://chinese-quartet.org/) and described in an accompanying paper by Yang et al. [46].

### Data generation, analysis, and normalization

Here, we provide a brief description of data generation, analysis, and normalization of transcriptomics, proteomics, and metabolomics data. Detailed description can be found in accompanying papers [41–46].

#### Transcriptomics

Transcriptomics datasets from the Quartet RNA reference materials were collected, consisting of 252 RNAseq libraries from 21 batches generated in eight labs using two library construction protocols (poly(A) selection and RiboZero) and two sequencing platforms (Illumina NovaSeq and MGI DNBSEQ-T7). Detailed information was described in the accompanying RNA paper [43].

RNAseq reads were aligned using HISAT2 and genes were quantified using StringTie followed by Ballgown [56]. Reference human genome build 38 (https://genome-idx.s3.amazonaws.com/hisat/grch38_snptran.tar.gz) and gene model from Ensembl (http://ftp.ensembl.org/pub/release-93/gtf/homo_sapiens/Homo_sapiens.GRCh38.93.gtf.gz) were used for read mapping and gene quantification. The read count and normalized data in Fragments Per Kilobase of transcript per Million mapped reads (FPKM) were obtained. A floor value of 0.01 was added to the FPKM value of each gene, and log2 transformation was then conducted.

#### Proteomics

Two batches of Quartet protein reference materials, in the form of dried tryptic peptide mixtures, were generated from the same batch of cultured cells. In this study, proteomics datasets from the first batch were collected, including 312 LC–MS/MS based profiling under a data-dependent acquisition mode (DDA) using different platforms and instruments at different labs. Detailed information was described in the accompanying protein paper [41, 44]. The MS platforms included Thermo Fisher Scientific™ Q Exactive™ hybrid quadrupole-Orbitrap™ series mass spectrometers (Q Exactive, Q Exactive Plus, Q Exactive HF and Q Exactive HF-X), Thermo Fisher Scientific™ Orbitrap Fusion™ Tribrid™ series mass spectrometers (Fusion and Fusion Lumos), Orbitrap Exploris 480 mass spectrometer, Sciex Triple-TOF 6600 and Bruker timsTOF Pro mass spectrometer.

MS raw files were searched against the National Center for Biotechnology Information’s (NCBI) human Refseq protein database (updated on 04–07-2013, 32,015 entries) using Firmiana 1.0 enabled with Mascot 2.3 (Matrix Science Inc)[57]. False discovery rate (FDR) by using a target-decoy strategy was set to 1% for both proteins and peptides. Proteins were then quantified using the label-free intensity-based absolute quantification (iBAQ) approach. The fraction-of-total (FOT) was used to represent the normalized abundance of a particular protein, which was defined as a protein’s iBAQ value divided by the total iBAQ of all identified proteins within one sample [57]. Missing values was treated using two strategies. On one hand, when dealing with raw data without batch correction and applying BECAs including batch mean centering (BMC), Harmony, surrogate variable analysis (SVA) and ratio-based scaling, we used zero to replace the missing values, because it is more frequently used [58]. On the other hand, because several BECAs cannot perform adjustment when a feature is uniformly expressed within a single batch, if missing values were replaced with zero, about 44% of proteins were not able to be properly corrected. Therefore, a random value approximately zero was used to replace the missing values when ComBat, RUVg and RUVs were applied. It was implemented using *rnorm* function with a mean of zero and a standard deviation of 0.01. A floor value of 0.01 was then added to the value of each protein, and log2 transformation was conducted.

#### Metabolomics

Quartet metabolite reference materials were established in the form of dried cell extracts. A total of 264 LC–MS/MS based profiling were generated from 22 batches in five labs. Non-targeted and targeted metabolomics profiling were then conducted. More information was detailed in the accompanying metabolite paper [45]. In brief, the non-targeted metabolomics datasets were generated using AB SCIEX Triple TOF 5600, AB SCIEX QTRAP 6500, AB SCIEX TripleTOF 6600, and Thermo Fisher Scientific Q Exactive HF hybrid quadrupole-Orbitrap mass spectrometer systems, while the targeted metabolomics datasets were generated using Waters Xevo TQ-S and AB SCIEX QTRAP 6500 mass spectrometers.

Raw data were extracted, peak-identified and QC processed using the in-house methods in each lab. Compound identification was conducted using in-house library based on the retention time/index (RI), mass to charge ratio (m/z), and MS spectral data for each metabolite. Metabolite quantification was conducted using area-under-the-curve or the concentration calculated by calibration curve using standards of each metabolite. Similar with treatment of proteomics data, we replaced the missing values with a random value approximately zero (using *rnorm* function with a mean of zero and a standard deviation of one) when applying ComBat, RUVg and RUVs, and with zero when dealing with raw data without batch correction and applying BECAs including BMC, Harmony, SVA and ratio-based scaling. A floor value of 1 was then added to the value of each metabolite, and log2 transformation was conducted.

### Full datasets, balanced subsets, and confounded subsets

Fifteen batches of transcriptomics, proteomics, and metabolomics data from different platforms, labs and with different data quality were employed and referred as full datasets in this study. In the full datasets, each batch comprised 12 libraries, consisting of 12 tubes with each representing one of the triplicates of a donor (D5, D6, F7 and M8). Therefore, 180 libraries (12 libraries per batch × 15 batches) were included in full datasets at each omics level. The full datasets were used for calculating intra-batch fold changes (FC) and differentially expressed features (DEFs).

We then employed a subset of datasets from the full datasets to create balanced and confounded scenarios for assessing the pros and cons of the BECAs. Here, we arbitrarily selected D6 as the common reference material, leaving the rest three as the study groups (D5, F7, and M8). In the balanced experiment scenario, one replicate was selected for each study group from each of 15 batches. This was done independently for each omics type. In the confounded experiment scenario, five batches were randomly assigned to each study group (D5, F7, or M8) for each omics type to extract all three replicates for the assigned study group. For both scenarios, all three replicates for the selected reference sample (D6) in each batch were retained for reference-sample-based BECAs. Therefore, 45 study samples and 45 reference samples in balanced and confounded scenarios were employed at each omics level. The experimental design ensured the consistent number of libraries included in the balanced and confounded scenarios, as well as the separation of study samples from the reference samples for objective evaluation of the impact of BECAs. Expression matrix of full datasets, balanced subsets, and confounded subsets were deposited in figshare [59].

### Batch-effect correction methods

#### Raw

Expression profiles without batch correction were defined as “raw” expressions.

#### Batch mean-centering (BMC)

Mean-centering per feature per batch is to set the mean of each feature across all the samples within each batch to zero. This approach is applied based on log2-transformed expressions.

#### ComBat/ComBat-seq

ComBat is one of the most popular BECA tools [27]. It applies empirical Bayes shrinkage to adjust the mean and the variance by pooling information across multiple genes for correcting batch-effects [30]. In addition, ComBat-seq extends ComBat adjustment framework to using negative binomial regression to estimate RNAseq count data [27]. The *ComBat* function in the sva 3.42.0 package [29] was implemented for normalized expressions of proteomics and metabolomics, while the *ComBat_seq* function in the ComBat-seq package [27] was implemented based on transcriptomics count data.

#### Harmony

Harmony uses an iterative clustering-correction procedure based on soft clustering to correct for sample differences. The algorithm first combines the batches and projects the data into a dimensionally reduced space using PCA, and then uses an iterative procedure to remove the batch effects. The *HarmonyMatrix* function in the harmony 0.1.0 package [47] was implemented, using default parameter settings.

#### Surrogate variable analysis (SVA)

SVA is able to remove unwanted sources of variation while protecting the contrasts due to the primary variables specified in the function call. The *sva* function in the sva 3.42.0 package [29] was implemented to detect and remove latent variables, using default parameter settings.

#### Remove unwanted variation (RUV)

RUV uses a subset of the data to estimate the factors of unwanted variation adjusts for nuisance technical effects. We applied two modes for estimating the factors of unwanted variation, including: (1) RUVg, using negative control genes, assumed not to be differentially expressed with respect to the covariates of interest; and (2) RUVs, using reference sample (D6) for which the covariates of interest are constant [28]. The *RUVg* and *RUVs* functions in the RUVSeq 1.28.0 package [28] was implemented, using default parameter settings.

#### Ratio-based scaling

Ratio-based scaling is to convert expression profiles to relative-scale profiles within each batch on a feature-by-feature basis. Ratio-based scaling data were obtained by subtracting log_2_-transformed expression profiles of a feature by the mean of log_2_-transformed expression profiles of the three replicates of reference sample (D6) in the same batch.

### Detected features

The number of original features was 58,395, 8,150, and 984 for transcriptomics, proteomics, and metabolomics, respectively. For transcriptomics, a gene was considered detectable if the FPKM value was equal or higher than 0.1 in over 30% of the libraries. For proteomics, a protein was considered detectable if the normalized FOT value was equal or higher than 0.1 in over 30% of libraries. For metabolomics, a metabolite was considered detectable if the normalized value was equal or higher than 1 in over 70% of libraries. Features that were detected in both balanced and confounded datasets were included for further analysis. After filtering, the number of features considered to be detected across multiple batches and used in further analysis in each omics type was 26,907, 3,489, and 71 for transcriptomics, proteomics, and metabolomics, respectively.

### Intra-batch differential expression

Intra-batch differential expressions, including intra-batch FC and DEF/non-DEF, were calculated based on full datasets which consisted of four donors with three replicates per donor in each batch.

Intra-batch FCs of three group-pairs (F7/D5, M8/D5, and M8/F7) were calculated. In each batch, the three replicates were first averaged and then the FCs were computed for three group-pairs. In order to improve the reliability of the FCs, features that were satisfied with thresholds of t-test *p* < 0.05 were used for further analysis.

Intra-batch DEFs/non-DEFs were then identified. According to recommendations from MAQC/SEQC Consortia [5, 60], a nonstringent t-test *p* value cutoff with a sufficient FC could be used to identify differentially expressed genes. In this study, a feature was considered as a DEF or non-DEF in each batch using the following criteria: up-regulated DEFs (student’s t-test *p* < 0.05 and FC > 2 for transcriptomics and proteomics or > 1.5 for metabolomics), down-regulated DEFs (*p* < 0.05 and FC < 0.5 for transcriptomics and proteomics or < 0.667 for metabolomics), and non-DEFs (the remaining features).

### Reference datasets of differential expression

Reference datasets of differential expressions, including reference FC and DEF/non-DEF, were constructed based on a consensus-based strategy and could be used as “ground truth” for benchmarking.

The reference FC between each pair of donors for a feature was provided in the format of an average over the 15 intra-batch FCs. The reference FCs were retained for those features that had t-test *p* < 0.05 in at least three batches across the two donors.

A list of reference DEFs and non-DEFs were then identified based on intra-batch DEFs and non-DEFs using voting for consensus. Specifically, within each batch in each group-pair, a feature can be classified into one of the three groups, i.e. up-regulated DEF, down-regulated DEF and non-DEF. The choice with the most first-preference votes from 15 votes from 15 batches is the final classification of the feature. Features receiving more than one first-preference vote were not included in reference datasets.

### Cross-batch differential expression

Cross-batch differential expressions, including FC and DEF/non-DEF, were calculated based on subset datasets in balanced and confounded scenarios. In each comparison in balanced scenario, to ensure that three replicates of each donor were included, three batches of datasets were randomly selected in the balanced scenario. For example, three replicates of D5 from batch X, Y and Z were compared with three replicates of F7 from batch X, Y and Z. Cross-batch FC and DEF/non-DEF were further calculated. On the other hand, in confounded scenario, as each batch included three replicates in one donor, one batch of each donor was randomly selected. For example, three replicates of D5 from batch X were compared with three replicates of F7 from batch Y. Similar methods and criteria with intra-batch FC and DEF/non-DEF were used for calculating cross-batch FC and DEF/non-DEF. In each group-pair, the process was repeated 15 times for eliminating potential selection bias.

### Prediction models

Frequently, a predictive model was built using some dataset(s), and was further validated using independent dataset(s). These datasets were probably confounded with batch effects. To simulate the clinical context, age and sex corresponding to the donor of each library were used as biological endpoints to assess the reliability of cross-batch prediction.

Prediction models were developed and validated using a two-layer validation strategy [3]. Briefly, datasets were first divided into two sets, comprising 27 libraries from nine batches as training set, and the remaining 18 libraries from six batches as validation set, according to data generation date. The training set was then used to select variables and train prediction models using five machine-learning algorithms, including model averaged neural network (avNNet), support vector machine (SVM), random forest (RF), generalized partial least squares (GPLS), and linear algorithm BstLm, through an internal-layer of 25 runs of fivefold cross-validation process to resist overfitting. The models were further validated using the validation set as an external-layer evaluation. The *train* and *predict* functions in the caret 6.0.90 package were implemented, using default parameter settings (https://github.com/topepo/caret).

### Integration of multiomics data

Expression profiles from 36 samples of three donors derived from 12 batches in each omics type were randomly selected from the dataset (*N* = 45) and further used for multiomics integration. In order to eliminate selection bias, this process was repeated ten times. Three integrative tools were used, including iClusterBayes from iClusterPlus 1.30.0 package [61], intNMF from IntNMF 1.2.0 package [49], and SNF from SNFtool 2.3.1 package [48]. The number of eigen features of iClusterBayes was set to 3. The number of clusters of IntNMF was set to 3. Parameters for SNF were set as follows: the number of neighbors package (12), hyperparameter (0.5), the number of iterations (10), and the number of clusters (3). All other parameters were set by default.

### Performance evaluation

#### Signal-to-noise ratio (SNR)

SNR is defined as the ratio of the average distance among different donors (*e.g.* D5-1 vs F7-1) from the average distance among technical replicates of the same (*e.g.* D5-1 vs D5-2). Based on principal component analysis (PCA), distances of two samples in the space defined by the first two PCs were used to represent distances between the two samples. SNR was calculated as Eq. 1:

1 $$SNR=10\times {log}_{10}\left(\frac{m\times \left(\genfrac{}{}{0pt}{}{n}{2}\right)}{\left(\genfrac{}{}{0pt}{}{m}{2}\right)\times n\times n}\times \frac{\sum_{x=1}^{m}\sum_{y=x+1}^{m}\sum_{i=1}^{n}\sum_{j=1}^{n}\sum_{p=1}^{2}{W}_{p}(P{C}_{p,i,x}-P{C}_{p,j,y}{)}^{2}}{\sum_{x=1}^{m}\sum_{i=1}^{n}\sum_{j=i+1}^{n}\sum_{p=1}^{2}{W}_{p}(P{C}_{p,i,x}-P{C}_{p,j,x}{)}^{2}}\right)$$

where $m$ is the number of donors, and $n$ is the number of replicates in each donor.${W}_{p}$ represents the p^th^ principal component of variances.$P{C}_{p,i,x},P{C}_{p,j,x}$ and $P{C}_{p,j,y}$ represent the p^th^ component values of replicate $i$ and replicate $j$ in donor $x$ or donor $y$, respectively.

#### Evaluation based on differential expression

We then developed several quality metrics, including, relative correlation (RC) of FCs and MCC of DEFs, for the evaluation of BECA methods in terms of differential expression.

RC was calculated based on the Pearson correlation coefficient between the FCs for a given pair of donors and the corresponding reference FC values. It is referred to as the “relative correlation” metric, representing the numerical consistency with the “ground truth”. To improve reliability, the mean of the three replicates of each donor was calculated before performing ratio-based expression analysis. FC were transformed using log2 scaling.

Moreover, we compared cross-batch DEFs with reference DEFs and non-DEFs, and calculated the number of true positives (TP), true negatives (TN), false positives (FP), and false negatives (FN). Matthews Correlation Coefficient (MCC) was further calculated to measure the consistency of DEFs detected from cross batches for a given group-pair with “ground truth”. This metric is called the “MCC of DEFs”. MCC was computed using the Eq. 2. Furthermore, typical performance metrics, including sensitivity, specificity, precision was calculated using Eqs. 3, 4 and 5, respectively. Furthermore, Jaccard index of DEFs was introduced to compare DEFs identified from cross batches with reference datasets to see which features were shared and which were distinct, representing similarity of the cross-batch DEFs with reference DEFs.

2 $$MCC=\frac{\mathrm{TP}\times \mathrm{TN}-\mathrm{FP}\times \mathrm{FN}}{\sqrt{(\mathrm{TP}+\mathrm{FP})(\mathrm{TP}+\mathrm{FN})(\mathrm{TN}+\mathrm{FP})(\mathrm{TN}+\mathrm{FN})}}$$

3 $$Sensitivity=\frac{\mathrm{TP}}{\mathrm{TP}+\mathrm{FN}}$$

4 $$Specificity=\frac{\mathrm{TN}}{\mathrm{TN}+\mathrm{FP}}$$

5 $$Precision=\frac{\mathrm{TP}}{\mathrm{TP}+\mathrm{FP}}$$

In each comparison, three replicates of each donor were included, making them suitable for statistical analysis. Specifically, three batches (each batch contained one replicate of a donor) of datasets were randomly selected in balanced scenario, while one batch (each batch contained three replicates of a donor) from each of two donors under comparison was randomly selected in confounded scenario. Cross-batch RC and MCC of DEFs between cross-batch values with reference values were then computed. To eliminate potential selection bias, this process was repeated 15 times. Moreover, since three comparisons were possible, the RC was calculated in each comparison. A total of 45 (15 repeats × 3 group-pairs) RC and MCC of DEFs values under each BECA method in each scenario and each omics type were obtained. Mean with standard error (s.e.) of RC and MCC of DEFs were further calculated, representing performance of BECAs in identifying differential expression.

Moreover, the primary goal of BECAs is to make cross-batch expression profiles like intra-batch expression profiles. Hence, intra-batch RC and MCC of DEFs were used as the positive controls and were calculated as the correlation values between intra-batch values with the reference values. However, due to lab proficiency and/or technical limitations, variations existed in each batch and led to variations in intra-batch values across 15 batches. Hence, intra-batch RC and MCC of DEFs were not equal to 1.

#### Evaluation based on prediction models

Model performances were assessed using multiple performance metrics, including F1 score, MCC, sensitivity, specificity, Pos.Pred.Value (positive prediction value), Neg.Pred.Value (negative prediction value), precision, and accuracy. Model construction and assessment was implement using caret package 6.0.90 (https://github.com/topepo/caret).

#### Adjusted Rand Index (ARI)

The number of true groups was set to three, with one group corresponding to the multiomics samples of the same donor. The Adjusted Rand Index (ARI) was used to measure consistency of clustering after multiomics integration with true group labeling. The Rand Index (RI) computes a similarity measure between clusters by considering all pairs of samples and counting pairs that are assigned in the same or different clusters in the predicted and true clusters. The raw RI score is then “adjusted for chance” into the ARI score as follows:

$$ARI=\frac{RI+E\left(RI\right)}{max\left(RI\right)-E\left(RI\right)}$$

### Simulation of low-quality reference samples

We used two strategies for simulation of low-quality reference samples to evaluate the consistency of ratio-based approach under different data quality scenarios. First, we artificially introduced different levels of noise to the reference samples to evaluate the consistency of ratio-based approach under different data quality scenarios. Specifically, for each feature in each batch, we randomly generated three values of “modified” reference samples using *rnorm* function in R package with mean zero and SD equal to different folds of SD that were calculated from three replicates of reference samples in the batch. Different folds of SD ranging from 0.1 to 10 were used to mimic different levels of noises in the reference. Ratio-based method was further evaluated using the average of the three “modified” replicates of reference samples as the denominator.

Secondly, we artificially reduced the expression level of some genes to simulate reference samples with low-quality in transcriptomics data. For each replicate of the reference sample, we randomly selected a certain percentage of genes and artificially reduced their read counts to 1/2, 1/4, or 1/8 of the original levels. Using this simulation strategy, the affected genes across different replicates in the same batch were probably different, which was as expected. Modified read counts in each library were then normalized to Count Per Million (CPM). Ratio-based profiles were further conducted based on log2-transformed CPM using the average of the three replicates of reference samples as the denominator. 

### Statistical analysis and data visualization 

All statistical analyses and data visualization were implemented using R statistical packages 4.1.2 (https://www.r-project.org). Student’s t-test was used to compare continuous variables. PCA was conducted with the univariance scaling, using the *prcomp* function. tSNE was conducted using the R package Rtsne 0.15. Data visualization was implemented using the R packages ggplot2 3.3.5 (https://ggplot2.tidyverse.org/), GGally 2.1.2 (http://ggobi.github.io/ggally/), and ggsci 2.9 (https://github.com/nanxstats/ggsci).

### Supplementary Information

**Additional file 1: Fig. S1.** Diversity of quality of original datasets. **Fig. S2.** tSNE plots based on different batch-effect correction methods. **Fig. S3.** Workflow of construction of reference fold change and reference differentially expressed features. **Fig. S4.** Evaluation of the performances of BECAs using sensitivity, specificity, precision, and Jaccard Index of identification of differentially expressed features. **Fig. S5.** Matthews Correlation Coefficient of identification of differentially expressed features using with or without controlling multi-testing methods. **Fig. S6.** Evaluation of the performances of BECAs based on model prediction results. **Fig. S7.** SNR of the ratio-based method under different data quality scenarios by introducing different levels of noise to the reference samples. **Fig. S8.** SNR of the ratio-based method under different data quality scenarios by artificially reducing expression level of some genes in the reference samples.

**Additional file 2.** Review history.

## Data Availability

The raw sequence data reported in this paper have been deposited in the Genome Sequence Archive (GSA) (accession number: HRA001859) [62]. Moreover, we have developed the Quartet Data Portal [46] for the community to access and share the Quartet multiomics resources. The expression profiles used in the manuscript, including full datasets and datasets of balanced and confounded scenarios, have been deposited in figshare [59].
