# Supplementary material for: Correcting batch effects in large-scale multiomics studies using a reference-material-based ratio method
Source: Genome Biol. 2023 Sep 7;24:201. doi: 10.1186/s13059-023-03047-z (PMC10483871; doi:10.1186/s13059-023-03047-z)
Supplement: Supplementary file 1 — Additional file 1: Fig. S1. Diversity of quality of original datasets. Fig. S2. tSNE plots based on different batch-effect correction methods. Fig. S3. Workflow of construction of reference fold change and reference differentially expressed features. Fig. S4. Evaluation of the performances of BECAs using sensitivity, specificity, precision, and Jaccard Index of identification of differentially expressed features. Fig. S5. Matthews Correlation Coefficient of identification of differentially expressed features using with or without controlling multi-testing methods. Fig. S6. Evaluation of the performances of BECAs based on model prediction results. Fig. S7. SNR of the ratio-based method under different data quality scenarios by introducing different levels of noise to the reference samples. Fig. S8. SNR of the ratio-based method under different data quality scenarios by artificially reducing expression level of some genes in the reference samples. [file 13059_2023_3047_MOESM1_ESM.pdf]

**Additional file 1: Supplementary figures**

# Correcting batch effects in large-scale multiomics studies using a reference-material-based ratio method

Yu Y et al.

*Genome Biology*

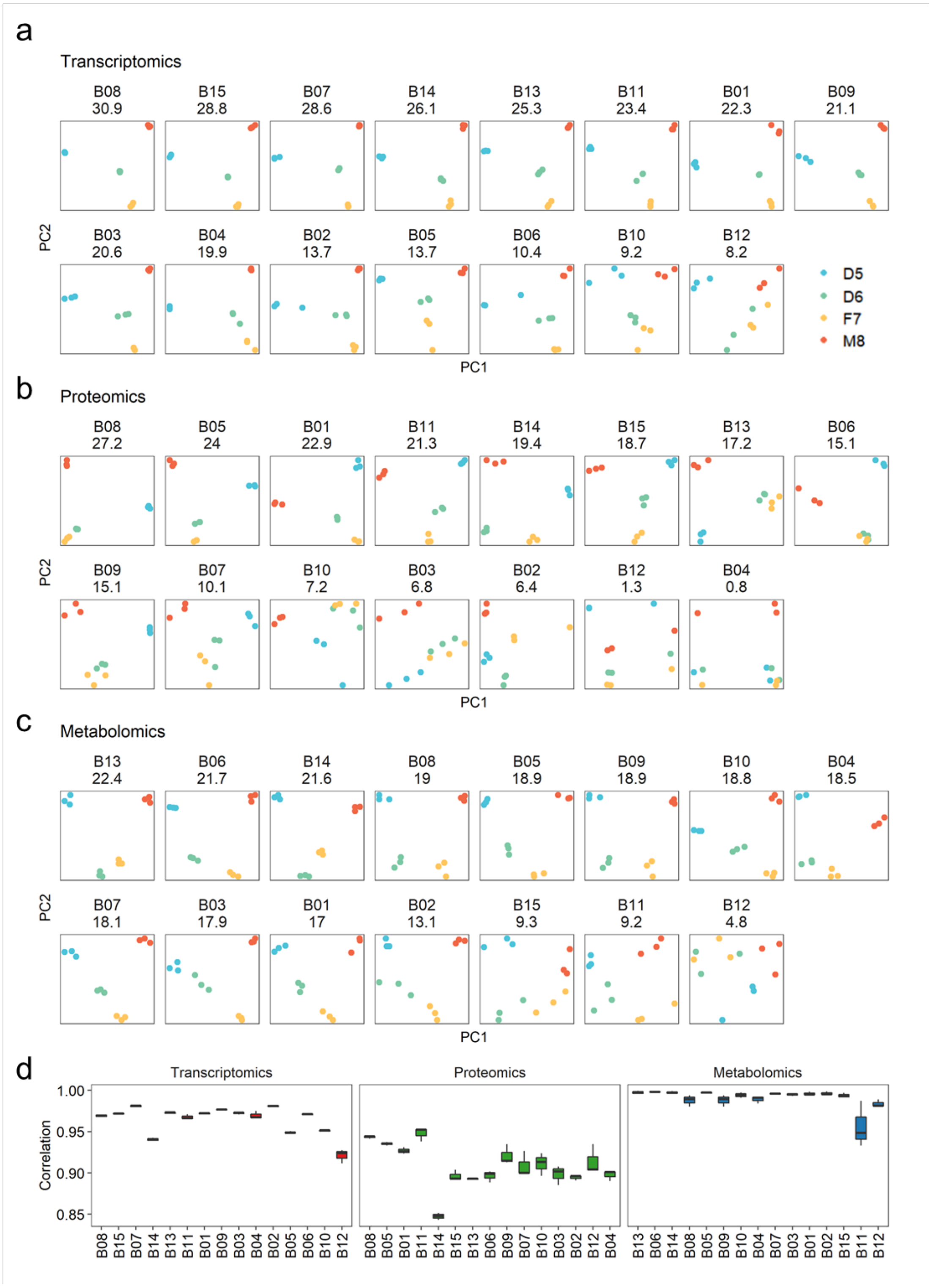


**Fig. S1 | Diversity of quality of original datasets.**

(a-c) PCA plots per batch ordered by the SNR value for datasets in transcriptomics (a), proteomics (b) and metabolomics (c). (d). Pearson correlation coefficient of replicates of reference sample (D6) in each batch.

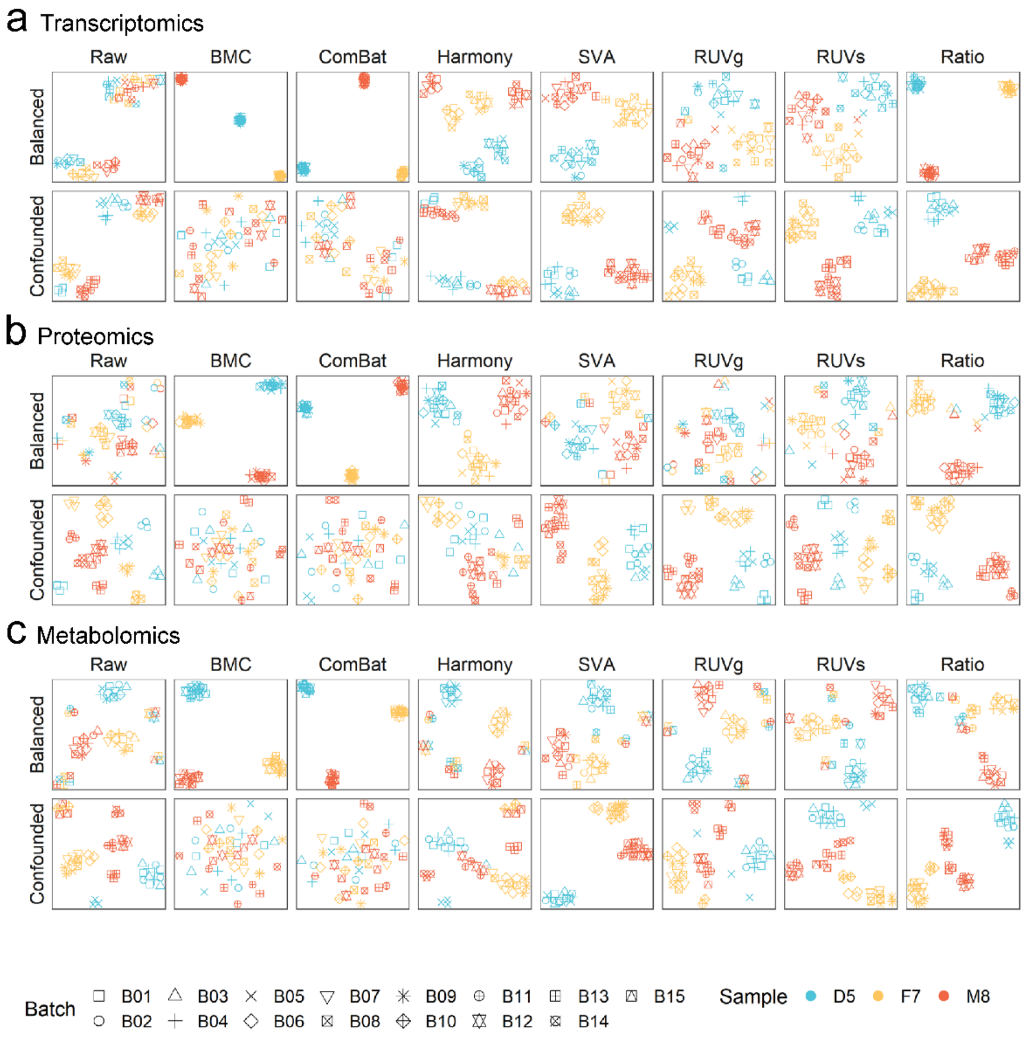


**Fig. S2 | tSNE plots based on different batch-effect correction methods.**

tSNE plots based on different batch-effect correction algorithms (BECAs) in balanced and confounded scenarios, using (**a**) transcriptomics, (**b**) proteomics, and (**c**) metabolomics data. Plots were color-coded by donor (D5, F7, and M8) and shaped by batch.

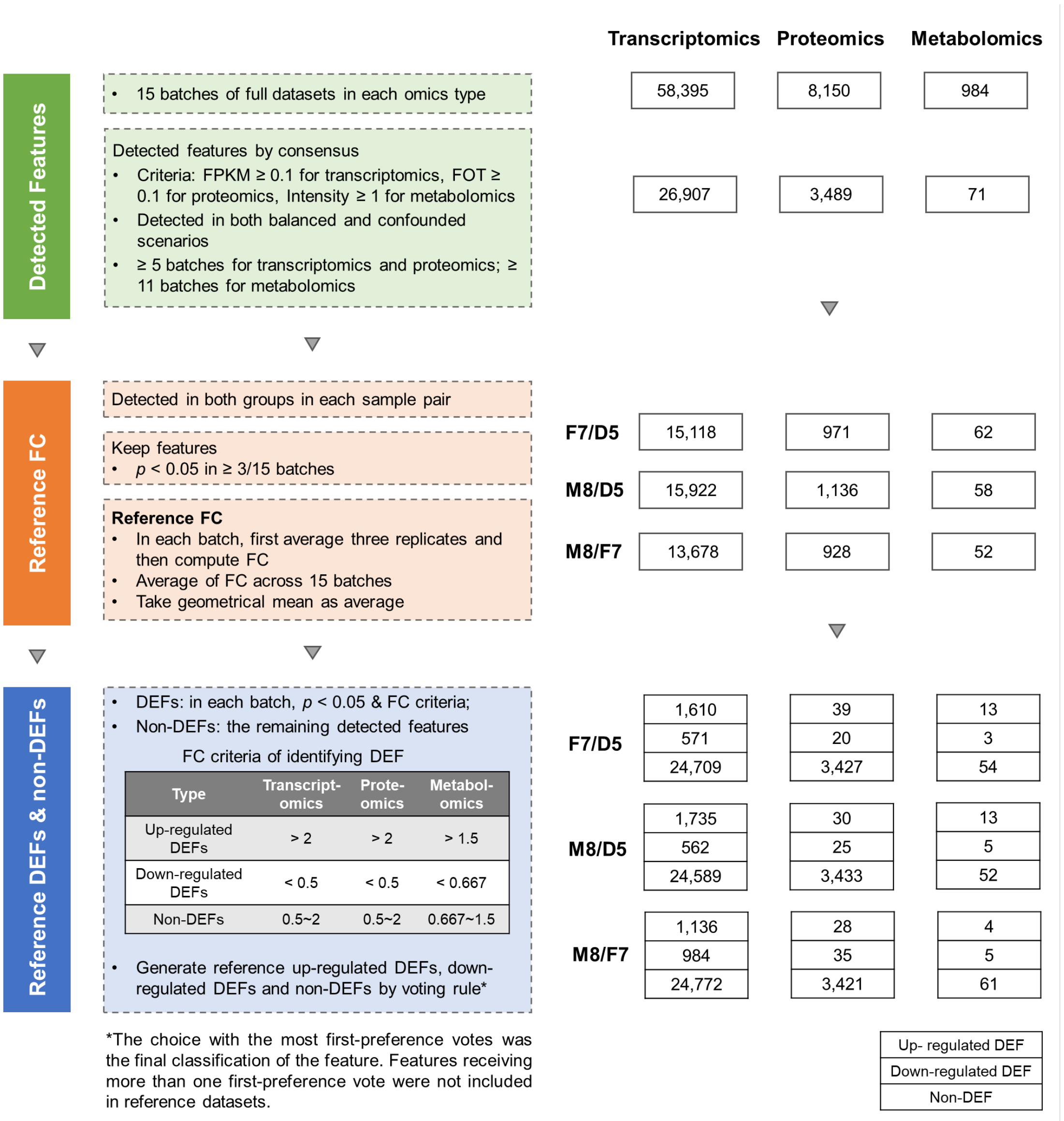


**Fig. S3 | Workflow of construction of reference fold change and reference differentially expressed features.**

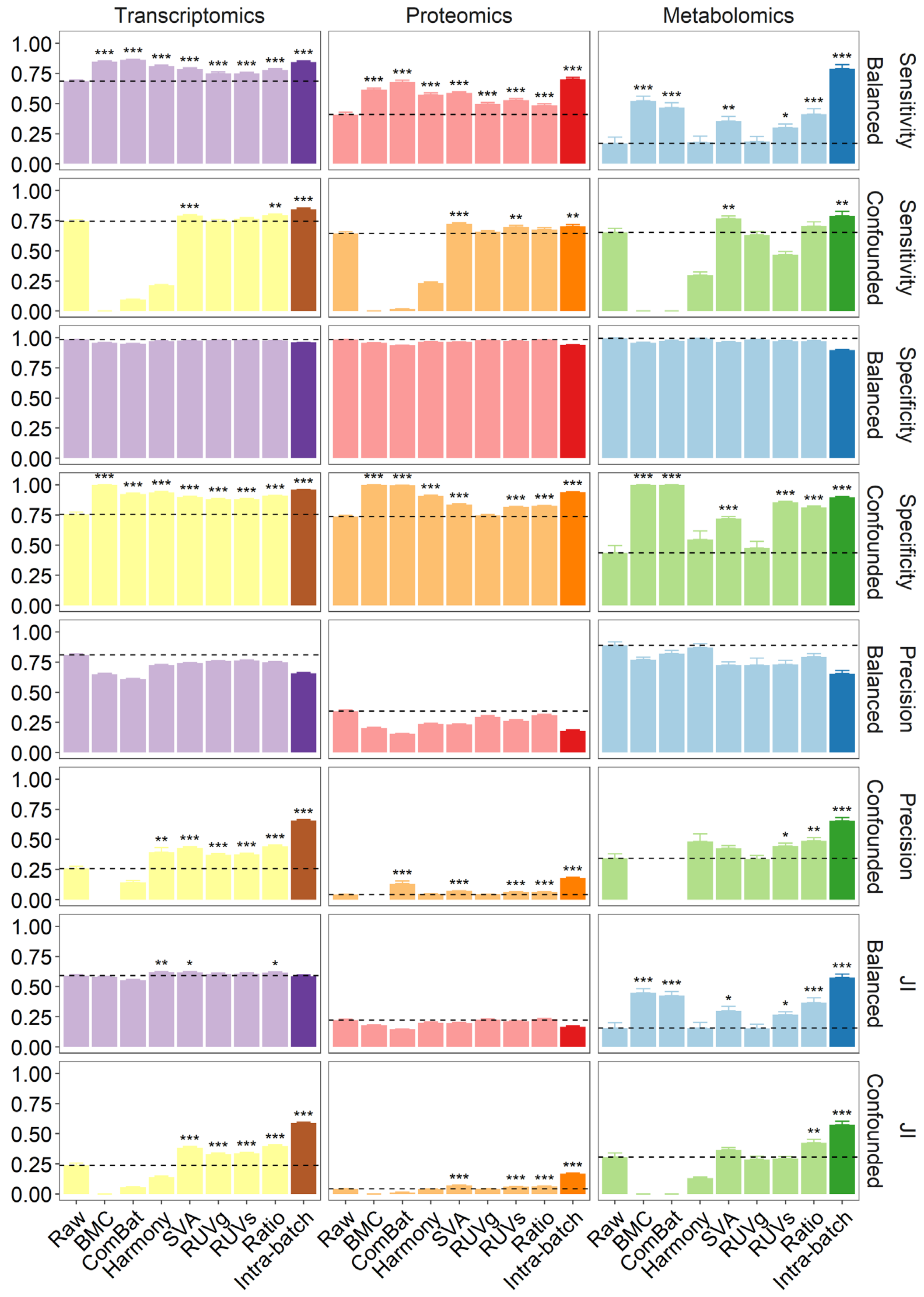


**Fig. S4 | Evaluation of the performances of BECAs using sensitivity, specificity, precision, and Jaccard Index of identification of differentially expressed features.**

Bar plots (mean ± s.e.) representing sensitivity, specificity, precision, and Jaccard Index (JI) of differentially expressed features with reference datasets across seven

BECAs were conducted in balanced and confounded scenarios using transcriptomics, proteomics, and metabolomics data. Metrics based on intra-batch DEFs were calculated based on full datasets and used as positive controls. Mean value of the dataset without correction (raw) in each panel was plotted in dashed line. Performances between the raw group and BECA groups were compared using Student's t-test. A group with the performance significantly higher than raw group was marked with stars (*). Symbolic number coding of *p*-value was used as: *** ($p \leq 0.001$), ** ($0.001 < p \leq 0.01$), * ($0.01 < p \leq 0.05$).

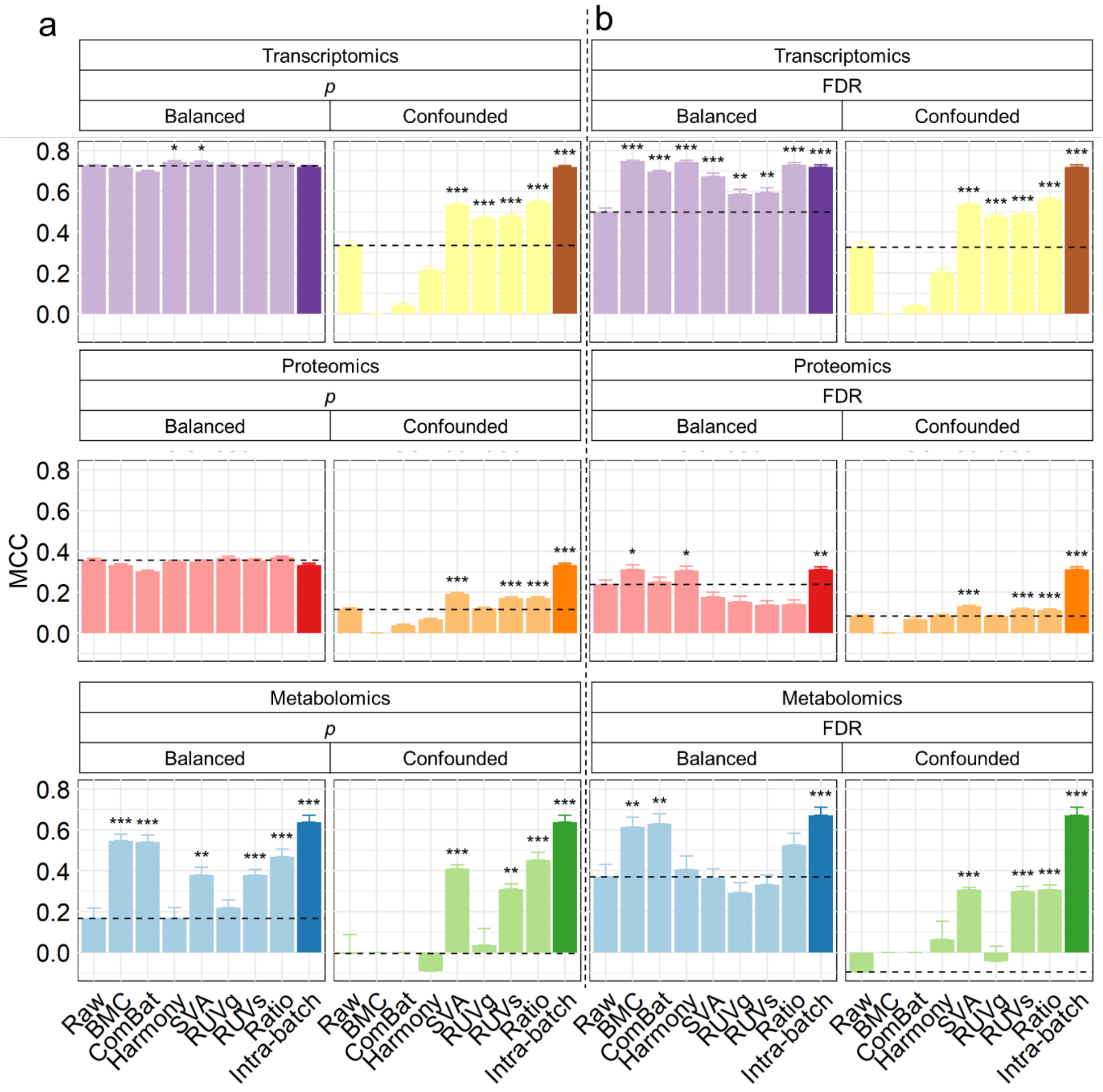


**Fig. S5 | Matthews Correlation Coefficient of identification of differentially expressed features using with or without controlling multi-testing methods.**

Cutoff of identification of differentially expressed features (DEFs) was set as: (a) $p$ < 0.05 and FC > 2 or < 0.5 for transcriptomics and proteomics and > 1.5 or < 0.667 for metabolomics; (b) FDR < 0.05 and FC > 2 or < 0.5 for transcriptomics and proteomics and > 1.5 or < 0.667 for metabolomics. Matthews Correlation Coefficient (MCC) of DEFs with reference datasets were used as a metric.

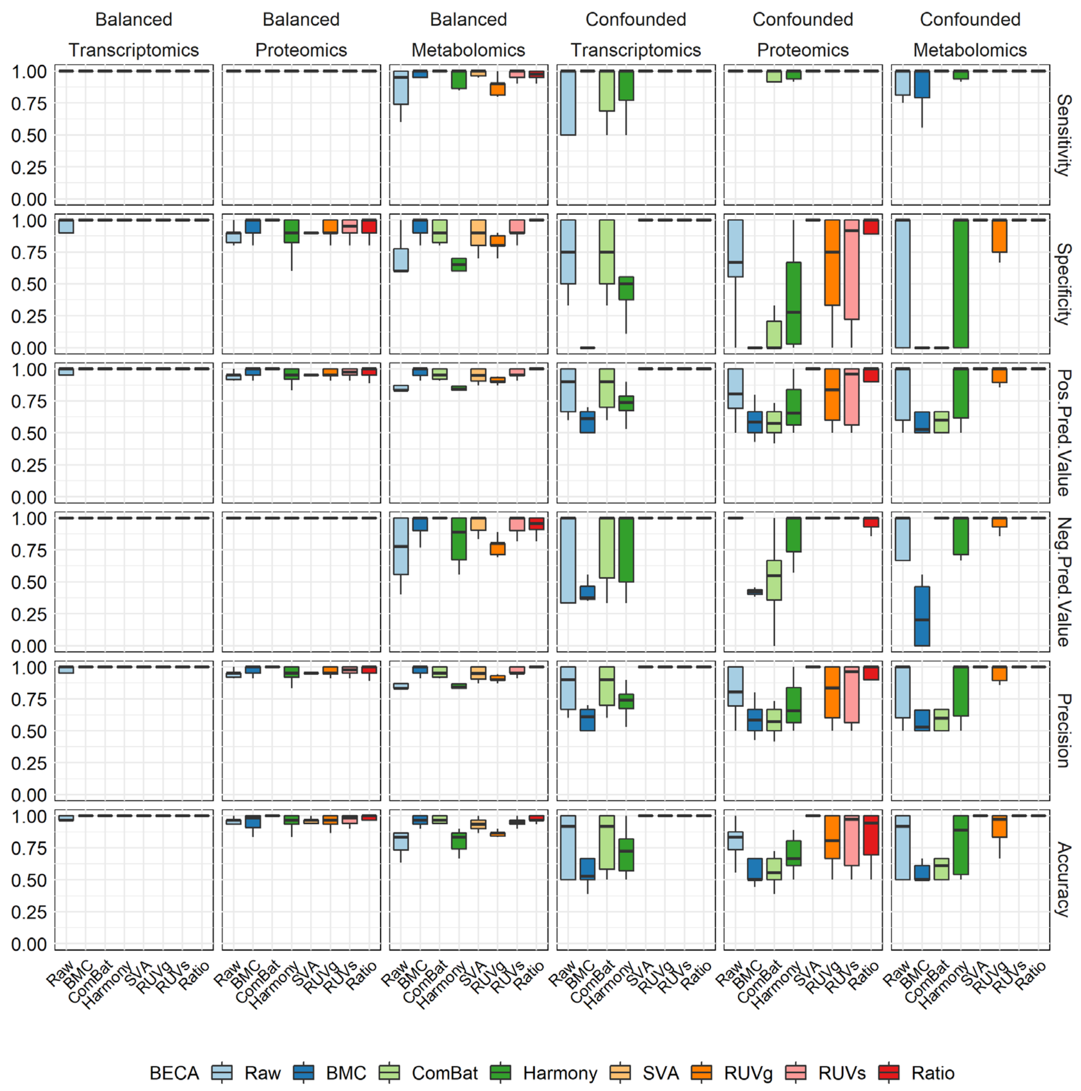


**Fig. S6 | Evaluation of the performances of BECAs based on model prediction results.**

Validation performances in predicting sex and age using five machine-learning algorithms under balanced and confounded scenarios. Model performances were measured using sensitivity, specificity, Pos.Pred.Value (positive prediction value), Neg.Pred.Value (negative prediction value), precision, and accuracy.

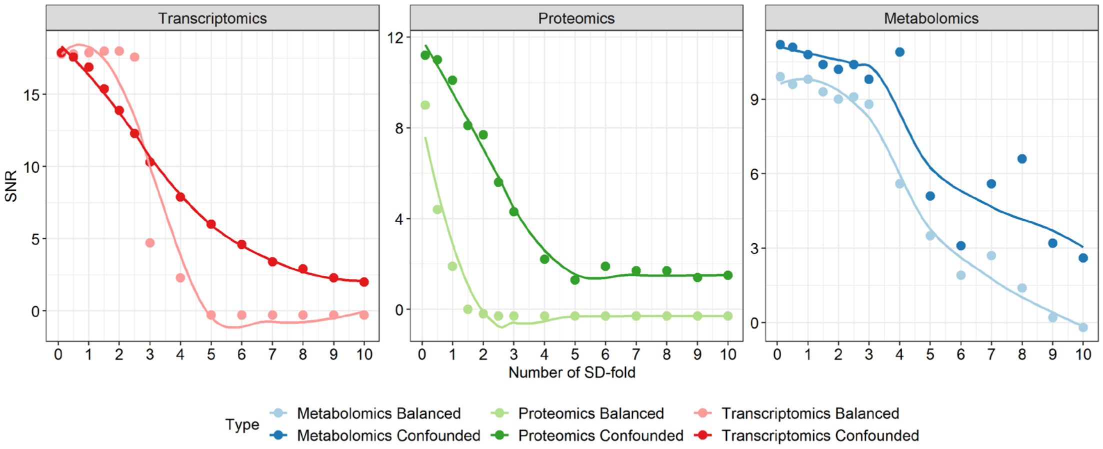


**Fig. S7 | SNR of the ratio-based method under different data quality scenarios by introducing different levels of noise to the reference samples**

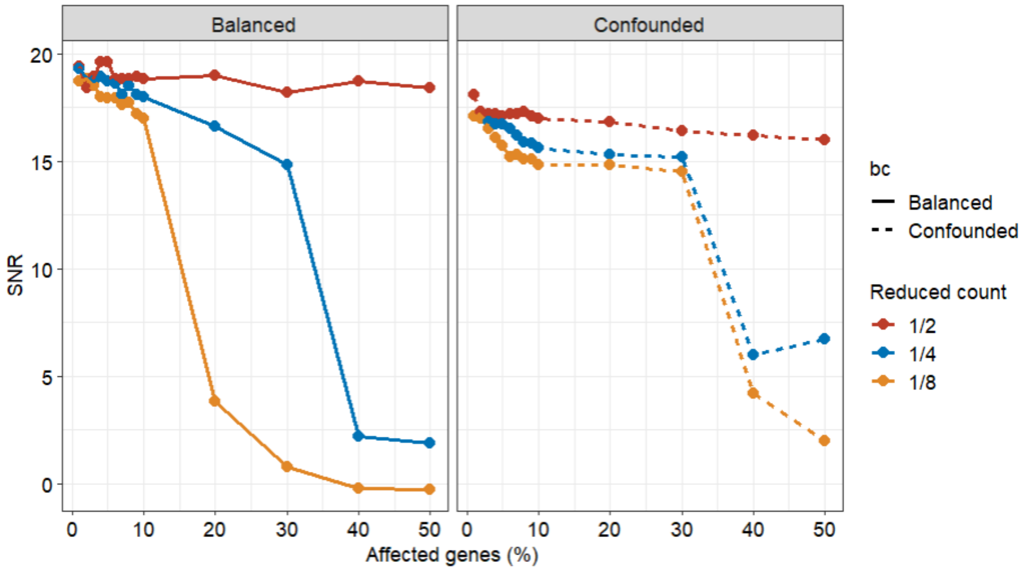


**Fig. S8 | SNR of the ratio-based method under different data quality scenarios artificially reducing expression level of some genes in the reference samples**
