# Supplementary material for: Correcting batch effects in large-scale multiomics studies using a reference-material-based ratio method
Source: Genome Biol. 2023 Sep 7;24:201. doi: 10.1186/s13059-023-03047-z (PMC10483871; doi:10.1186/s13059-023-03047-z)
Supplement: Supplementary file 2 — Additional file 2. Review history. [file 13059_2023_3047_MOESM2_ESM.docx]

Review History

**First round of review**

**Reviewer 1**

**Were you able to assess all statistics in the manuscript, including the appropriateness of statistical tests used?** Yes, and I have assessed the statistics in my report.

**Comments to author:**

In this submission, Yu et al. compare various batch effect correction methods on 3 types of omics data. Because batch effect can be a major source of variation in omics data, it is important to understand its impact on scientific results. Interestingly, the authors use standard analysis types such as sample clustering, differential analysis and predictive modelling to demonstrate better results after some batch effect correction methods. Also interesting is the experimental design composed of specific batches of samples representing balanced or confounded distributions of biological groups. Unfortunately, the experimental design has major limitations and the current submission does not describe its methods clearly enough to understand exactly the statistical procedure. Although shown to perform the best, their own method has serious limitations that must be more extensively discussed, especially as better implementations of reference material have been already published two decades ago. Therefore, this work may be better described as a proof of concept.

MAJOR POINTS

* The "universal reference" cell line is actually from a single female individual and a single cell type. This is by definition not universal. For example, it is not appropriate to normalize data from other cell types (what about cell-specific gene and protein expression?), from male samples (what about the Y chromosome?) and actually not appropriate to normalize any other sample that would likely have some common genomic differences (deletions or re-arrangement). For a better implementation of a universal reference, one would refer to different works published back in the 2000s (among others: Novoradovskaya et al. 2004, https://doi.org/10.1186/1471-2164-5-20; Puskás et al. 2002, https://doi.org/10.2144/02334mt03). The authors should discuss extensively those limitations in their manuscript.

* The need to include several technical reference samples in each batch that would have no other scientific purpose except normalization also represents a critical problem for a potential broad usage of this method. For instance, corresponding techniques proposed for microarrays did not became popular in the community.

* The idea to use such an elaborated experimental design composed of many batches is excellent, but in the manuscript the methods are not described enough to understand exactly the analyses. For example, the reviewer can not understand how statistical tests are done. It is also very difficult to follow what and why different subsets of the batches are used across the different analyses. The predictive modelling is also quite cryptic: e.g. what are the features? Overall, the manuscript should be substantially edited to better explain the methods with enough details to allow understanding and reproduction. Among other examples, it may help to make clear the definition of replicate and sample and to avoid using the word "sample" for different meanings (e.g. substitute "sample" to "donor" when appropriate?)

* About statistical tests, given the balanced scenario, I understand that each batch has only 1 replicate per human donor thus t-tests are not possible with batches and across 2 batches. Also, t-tests are not considered the most appropriate to derive differential genes from RNA-seq with small groups. Do the authors control multi-testing?

* The performance in DEF identification using the consensus of intra-batch DEFs should be also better explained. It is not clear what is done with the confounding subsets, as they will not have intra-batch comparisons. As authors consider the intra-batch consensus of DEF as ground truth, they should encourage researchers to produce such ground truth, instead of using statistical means to correct the data.

MINOR POINTS

* "These two endpoints (age and sex) are easy to predict": please include a citation to support this statement and write usual predictive performance for those tasks

* "we found that under the balanced scenario, the prediction performance of a machine learning method was equally good, regardless of the choice of BECA methods": It should be made clear here, that the classifiers trained non-corrected data still performed on par with the others.

**Reviewer 2**

**Were you able to assess all statistics in the manuscript, including the appropriateness of statistical tests used?** Yes, and I have assessed the statistics in my report.

**Comments to author:**

Yu and colleagues present an assessment of batch correction algorithms applied to three different types of omics modalities. The use the Quartet dataset, that contains multiple batches of the same set of four samples, each batch containing three technical replicates. Seven different BECAs were evaluated using different metrics. Scenarios of balanced and confounded designs are included. They concluded that the ratio-based approach, where a reference sample is used, is the best strategy to combine omics datasets processed in batches.

This is a nicely conducted and relevant study that might be useful to experimentalists that are expecting to perform experiments in batch. However, I feel that the conclusions of the study are biased towards the type of dataset used to evaluate the BECAS (a well designed experiment that involved creating many batches of a defined collection of samples). Moreover, some of the evaluation strategies are not clearly explained and important details, such as how many DEF were considered to drive conclusions, are not included. Also, discussion should be extended to include additional limitations of their approach. My specific comments are below:

1. Figure 3 is confusing. It shows the performance of different BECAs with the RC and MCC metrics, which are both correlation values in the fold-changes of DEGs of the BECA method with the ground-truth obtained as the consensus of DEG in the intra-batch analysis. However, the graphs include a bar for the intra-batch analysis, which is puzzling and this should be a self-correlation. The methodology should be better explained.

2. Also, the authors mention that their correlation metrics show better the differences between BECAs than typical performance metrics such as sensitivity and precision, but do not explain why. We know that correlation is a metric that can be biased by extreme values. Is this having an influence in this observation? In any case, an explanation of the difference between evaluation by correlation metrics and the traditional ways of performance evaluation should be provided.

3. The authors state that the ratio-based methods are the best solution for batch correction. I believe that the limitations of this approach should also be highlighted. For example, does the ratio work well regardless the quality of the reference samples? One way to test this is to repeat their analysis taking other samples as reference and evaluating the consistency of the conclusions, or by artificially introducing different noise levels in the references. Moreover, a reference-based method is possible when the introduction of a reference sample can be decided as part of the experimental design. However, this is not always the case of the analysis scenarios that require batch correction. For example, when combining already existing dataset a reference sample may not exist or be possible (i.e. when only two experimental groups are part of the study). Moreover, the method assumes that there is no interaction batch-reference sample. Does this always hold? All these aspects should be discussed, and additional analysis should be included to evaluate the consistency of the reference approach in different data quality scenarios.

4. The authors should also bring to the discussion some aspects indicated in the introduction, such as that previous BECAs evaluations provided contradictory results. Why is their study better or how this work sheds lights into previous discrepancies? Where other analysis scenarios considered in the previous studies?

5. I am puzzled about the definition of high confidence DEF and non-DEF. DEF should be found as such in at least 15% of the batches, while non-DEF should not be found as DEG in more than 50% of the batches. Therefore, a feature that is DEF in 20% of the batches and non-DEF in 80% of the batches will be both high confidence DEF and high confidence non-DEF? This makes no sense to me.

6. Also, the definition of intra-batch fold-changes could be improved. I assume that the fold-changes are calculated for each feature independently. As there are three replicates for each sample, are the replicates first averaged and then the fold changes computed, or is this done in a different way? Please note that the sentence starting half-way in line 532 is grammatically incorrect.

7. The computation of the relative correlation is also not clear. How are the multiple batches considered here? In the explanatory figure only 3 batches are shown. Do the RC calculations involve only three batches? Since three comparisons are possible, are the values averaged per feature or concatenated to compute correlations?

8. In the calculation of MCC of DEF. Are all batches included in the DEF analysis? Since different number of batches are available for different omics, does this affect the capacity for detection DEF? The authors should reveal how many DEF and non-DEF for each omics type were obtained in the grown-truth and in the evaluation of each BECA method. Readers must understand the numbers of features used to base conclusions.

9. In the calculation of the ARI, it is unclear what the true clusters are. Are the authors assuming that one cluster corresponds to the multiomics samples of the same group? If so, it is to be expected that the same within-group similarity is maintained across omics layers? This is not obvious to me, and the assumptions of this analysis should be clearly stated and discussed.

10. Please include some extra information about the multiomics datasets. Number of features in each case and treatment of missing values. Although this might be described in the respective papers, the information should be included in this paper for clarity.

**Authors Response**

**Point-by-point responses to the reviewers’ comments:**

**Reviewer 1:**

In this submission, Yu et al. compare various batch effect correction methods on 3 types of omics data. Because batch effect can be a major source of variation in omics data, it is important to understand its impact on scientific results. Interestingly, the authors use standard analysis types such as sample clustering, differential analysis and predictive modelling to demonstrate better results after some batch effect correction methods. Also interesting is the experimental design composed of specific batches of samples representing balanced or confounded distributions of biological groups. Unfortunately, the experimental design has major limitations and the current submission does not describe its methods clearly enough to understand exactly the statistical procedure. Although shown to perform the best, their own method has serious limitations that must be more extensively discussed, especially as better implementations of reference material have been already published two decades ago. Therefore, this work may be better described as a proof of concept.

*Response: We greatly appreciate the reviewer’s recognition of the importance of better understanding the impact of batch effect on scientific results and the potential contribution of our work to the field. The reviewer’s constructive comments and suggestions are very helpful for us to improve our manuscript. We are sorry that the Methods of the manuscript were not described clearly enough, and have updated the manuscript according to the reviewer’s comments and suggestions. Moreover, limitations of the ratio-based method are extensively discussed in the revised manuscript. Detailed point-by-point responses and information on the revision of the manuscript are described below. We sincerely hope that the reviewer now finds the revised manuscript acceptable for publication in Genome Biology.*

MAJOR POINTS

* The "universal reference" cell line is actually from a single female individual and a single cell type. This is by definition not universal. For example, it is not appropriate to normalize data from other cell types (what about cell-specific gene and protein expression?), from male samples (what about the Y chromosome?) and actually not appropriate to normalize any other sample that would likely have some common genomic differences (deletions or re-arrangement). For a better implementation of a universal reference, one would refer to different works published back in the 2000s (among others: Novoradovskaya et al. 2004, https://doi.org/10.1186/1471-2164-5-20; Puskás et al. 2002, https://doi.org/10.2144/02334mt03). The authors should discuss extensively those limitations in their manuscript.

*Response: Thank you very much for the important comment and critique. We are sorry for the confusion. We have changed the word “universal” to “common” in the revised manuscript whenever possible to avoid further confusion. In our study, “universal” reference materials refer to the reference samples that are commonly and concurrently profiled along with study samples in each batch, serving as a crucial common ground for any follow-up analysis and comparison. We deeply appreciate the advantages of having truly “universal” reference materials with broad coverage (expression) of as many genes as possible, like the work from Novoradovskaya et al. and Puskás et al. mentioned by the reviewer. In fact, gene coverage was one of the main criteria for selecting reference RNA samples when we launched the MAQC-I study (Shi L et al., NBT 2006). We completely agree with the reviewer that in terms of gene coverage, our current work could be better described as a proof of concept. Consequently, as suggested by the reviewer, we have extensively discussed the limitations of the ratio approach for genes not expressed in the “common” reference sample, as we did in other accompanying manuscripts associated with the Quartet Project.*

*We used Quartet D6 as an example reference material for conducting ratio-based method for monitoring and correcting batch effects, which was indeed derived from a single female individual and a single cell type. As the reviewer mentioned, some features cannot be successfully corrected when using D6 as the denominator, for example, features that are not expressed in reference materials, including cell-specific features in numerator or male-specific feature when using a female material as dominator. However, the ratio-based method can successfully mitigate batch effect from numerous features when expressed in both the study samples (numerator) and reference material(s) (denominator). For example, using D6 (female) as a reference material is able to remove batch effects of non-sex-specific genes in F7 (male) (Figs. 2a-c in the revised manuscript). Therefore, although the ratio-based method has many limitations, it still has unique values, especially when batch effects are completely confounded with biological factors of study interests.*

*Following the reviewer’s comments and suggestions, we added text on the limitations of the ratio method as follows in the Discussion section in the revised manuscript (Lines 436-464). “While the ratio-based method performed favorably in both balanced and confounded scenarios, it is not free of limitations. First, some features cannot be successfully corrected, for example, features that are not expressed in the reference material, including cell-specific features in numerator or male-specific feature when using a female material as the dominator. However, ratio-based scaling can successfully mitigate batch effect from numerous features when expressed in both the study samples (numerator) and reference material(s). Secondly, a reference-based method is possible when the introduction of a reference sample can be decided as part of the experimental design. It is not applicable when combining already existing dataset as the reference sample may not exist or be possible. Thirdly, applying ratiobased scaling needs to include reference samples in each batch, which means additional cost even if limited. Take RNAseq as an example, users can apply two or three samples or per batch of 96 libraries for ratio-based expression profiling, maintaining a reasonable additional cost (3/(96-3)=3.2%) per batch of 93 study samples. Additionally, when well-established reference materials are used, such as Quartet multiomics reference materials, users can monitor batch quality based on predefined ‘ground truth’ in reference materials for proficiency testing and quality control, which can take full use of reference samples. Fourthly, the performance of the ratiobased scaling can be affected by the quality of the reference sample. If the quality of the reference sample is not good, the power of ratio-based method will be compromised. One possible solution is to use multiple (for example, two or three) replicates of reference samples and take the averaged value as the denominator. Finally, the ratio method assumes that there is no interaction between batch and the reference sample, that is, the reference sample is invariant across batches or times. Here, considering the concurrent availability of large amounts of the Quartet multiomics reference materials with demonstrated short- and long-term stability, we therefore recommend using the Quartet multiomics reference materials for monitoring and correcting batch effects. The limitations and caveats of our reference materials and the ratio approach warrant further investigations.”*

* The need to include several technical reference samples in each batch that would have no other scientific purpose except normalization also represents a critical problem for a potential broad usage of this method. For instance, corresponding techniques proposed for microarrays did not became popular in the community.

*Response: Thank you very much for the comment and concern. As the reviewer correctly pointed out there was a lack of adoption by the microarray community of common reference materials in gene-expression studies. Indeed, applying the ratio-based method needs to include common reference sample(s) in each batch, which means additional cost. However, it seems to us that the increased cost is limited compared to the potential gain. Take RNAseq as an example, users can apply two or three samples/replicates per batch of 96 libraries for ratio-based expression profiling, resulting in a reasonable additional cost of 3.2% (3/(96-3)). Additionally, when well-established reference materials are used, such as Quartet multiomics reference materials, users can monitor batch quality based on pre-defined “ground truth” in reference materials for proficiency testing and internal quality control, which can take full use of technical reference samples. This comment has been added into the Discussion in the revised manuscript (see our response to Question #1).*

*We would like to point out that it is a common and mandatory practice in clinical testing to use reference materials for quality control and quality assurance even though the number of parameters being measured is usually very small. In the research setting with omics technologies where the number of parameters being measured is exponentially increased and so is the level of difficulty in achieving accurate measurement, it would become natural to exercise caution and follow good practice in quality assessment of omics data, as described in the ISO/TS 22690 (Genomics Informatics – Reliability Assessment Criteria for High-throughput Gene-expression Data).*

* The idea to use such an elaborated experimental design composed of many batches is excellent, but in the manuscript the methods are not described enough to understand exactly the analyses. For example, the reviewer can not understand how statistical tests are done. It is also very difficult to follow what and why different subsets of the batches are used across the different analyses.

*Response: We are delighted that the reviewer liked our experimental design with many batches. We are very sorry for that the Methods in our manuscript were not described with enough details for readers to understand exactly how the analyses were performed. We have updated the Methods section and added detailed descriptions in the revised manuscript. Specifically, description of statistical tests of identifying differentially expression features can be found in Lines 678-685 in the revised manuscript. Additionally, description of datasets and subsets of batches that were used across different analysis have been provided, including overall description (Lines 593-615), assessing differential expression (Lines 702-713), training and validation sets for prediction modeling (Lines 721-723), and integration of multiomics data (Lines 732- 735). Illustration of the full datasets used in the study (Fig. 1b) has also been updated in the revised manuscript for better understanding the data used in analysis.*

*The predictive modelling is also quite cryptic: e.g. what are the features? Overall, the manuscript should be substantially edited to better explain the methods with enough details to allow understanding and reproduction. Among other examples, it may help to make clear the definition of replicate and sample and to avoid using the word "sample" for different meanings (e.g. substitute "sample" to "donor" when appropriate?)

*Response: Thank you very much for the comments. We are very sorry for the lack of clarity in the Methods of the manuscript. In this study, a feature in predictive modelling refers to a gene/protein/metabolite that can be used for predicting a biological endpoint. We carefully revised the Methods section with enough details and tried to provide sufficient information for readers’ understanding and reproduction of our analysis.*

*We are sorry for the confusion caused by the misuse of the word "sample" for different meanings in the manuscript. Here, the word “replicates” refers to replicates from the same sample group (donor), whereas the word “sample” is a general word which should be combined with other word, such as, “sample group”, “study sample”, “reference sample” and so on. We carefully read through the manuscript to replace “sample” to other more specific word/phrase, such as donor, library (sample that includes replicate(s) with-in and between sample groups), and study sample.*

* About statistical tests, given the balanced scenario, I understand that each batch has only 1 replicate per human donor thus t-tests are not possible with batches and across 2 batches. Also, t-tests are not considered the most appropriate to derive differential genes from RNA-seq with small groups. Do the authors control multi-testing?

* The performance in DEF identification using the consensus of intra-batch DEFs should be also better explained. It is not clear what is done with the confounding subsets, as they will not have intra-batch comparisons. As authors consider the intra-batch consensus of DEF as ground truth, they should encourage researchers to produce such ground truth, instead of using statistical means to correct the data.

*Response: When conducting statistical tests in the balanced scenario, three batches of datasets were randomly selected and combined. Therefore, three replicates (one from each of the three batches) of each sample group were included, making them suitable for statistical analysis. This process was repeated 15 times for eliminating potential selection bias. A total of 45 (15 repeats x 3 sample pairs) RC values and MCC of DEFs in each scenario and each omics type were obtained. Mean with standard error (s.e.) of RC and MCC of DEFs were further calculated, representing performance of BECAs in identifying differential expression. Detailed description of the Methods has been provided in the revised manuscript (Lines 702-713 for conduction cross-batch differential expression and Lines 779-790 for assessment using RC and MCC of DEFs).*

*It is still under debate whether Student’s t-test is appropriate in differential expression analysis in omics analysis such as RNAseq with small groups. The MicroArray and Sequencing Quality Control consortia developed guidelines for microarray and RNAseq data analysis (Shi L et al., Nature Biotechnology 2006, PMID 16964229; Su Z et al., Nature Biotechnology 2014, PMID 25150838; Salit M & Woodcock J, Nature Biotechnology 2021, PMID 34504343). According to the MAQC/SEQC study using reference RNA samples with technical replicates (Shi L et al., Nature Biotechnology 2006, PMID 16964229), and the accompanying rat toxicogenomic study (Guo L et al., Nature Biotechnology 2006, PMID 17061323) with biological replicates, it was found that fold-change ranking with a nonstringent t-test p value cutoff outperforms in identifying differentially expressed genes, using consistency across microarray platforms as the metric. The number of samples used for these two previously published studies is also relatively small (n = 5 and 3) and comparable to this study (n = 3). Moreover, the t-test is still one popular method for RNAseq (Husi H. Computational Biology 2019, PMID 31815396), including t-test analogical methods such as Cuffdiff and Cuffdiff2 (Trapnell C. Nature Biotechnology 2010, PMID 20436464).*

*In the manuscript, we used Student’s t-test p value without controlling multi-testing. Here, we use false-positive rate (FDR) and t-test p value for statistical analysis to compare the performances between with or without controlling multi-testing, respectively. The criteria for the identification of differentially expressed features (DEFs) were set as FDR < 0.05 or p < 0.05, and FC > 2 or < 0.5 for transcriptomics and proteomics and > 1.5 or < 0.667 for metabolomics. MCC is used as a performance metric for assessing reliability of identifying DEFs. The conclusion remains unchanged whether with or without controlling for multi-testing. That is, ratio-based method is found to be much more effective than others, especially in confounded scenarios (Additional file 1: Fig. S5). We have added the result in the revised manuscript (Lines 245-259).*

MINOR POINTS

* "These two endpoints (age and sex) are easy to predict": please include a citation to support this statement and write usual predictive performance for those tasks

*Response: Thank you for your suggestion. We include citations to support the statement and provided usual performance values for predicting those tasks in Lines 467-472 in the revised manuscript. “For example, the MAQC/SEQC Consortia comprehensively assessed the prediction performances of classification models from multiple analysis teams based on microarray and RNAseq datasets, and found that sex was the easiest to predict (MCC: 0.839-0.973) compared to clinically relevant endpoints (MCC: 0.129- 0.748) (Shi L et al., Nature Biotechnology 2010, PMID 20676074; Zhang W et al., Genome Biology 2015, PMID 26109056). Additionally, the accuracy of predictive models for distinguishing between young and old individuals was high (AUC: 0.95) (Harries LW et al., Aging Cell 2011, PMID 21668623).”*

* "we found that under the balanced scenario, the prediction performance of a machine learning method was equally good, regardless of the choice of BECA methods": It should be made clear here, that the classifiers trained non-corrected data still performed on par with the others.

*Response: Thank you for your comment. Yes, the classifiers trained with non-corrected data still performed on par with the others under the balanced scenario. We have updated the sentence in the revised manuscript (Lines 276-278) as “the prediction performance of a machine learning method was equally good with or without BECAs, and there were no differences in performance among the BECAs.”*

**Reviewer 2:**

Yu and colleagues present an assessment of batch correction algorithms applied to three different types of omics modalities. The use the Quartet dataset, that contains multiple batches of the same set of four samples, each batch containing three technical replicates. Seven different BECAs were evaluated using different metrics. Scenarios of balanced and confounded designs are included. They concluded that the ratio-based approach, where a reference sample is used, is the best strategy to combine omics datasets processed in batches.

This is a nicely conducted and relevant study that might be useful to experimentalists that are expecting to perform experiments in batch. However, I feel that the conclusions of the study are biased towards the type of dataset used to evaluate the BECAS (a well designed experiment that involved creating many batches of a defined collection of samples). Moreover, some of the evaluation strategies are not clearly explained and important details, such as how many DEF were considered to drive conclusions, are not included. Also, discussion should be extended to include additional limitations of their approach.

*Response: We greatly appreciate the reviewer’s positive assessment on the value of our work. The reviewer’s constructive comments and suggestions are very helpful to improve our manuscript. Details of the evaluation strategies as well as limitations of the ratio-based method are provided in the revised manuscript.*

My specific comments are below:

1. Figure 3 is confusing. It shows the performance of different BECAs with the RC and MCC metrics, which are both correlation values in the fold-changes of DEGs of the BECA method with the ground-truth obtained as the consensus of DEG in the intra-batch analysis. However, the graphs include a bar for the intra-batch analysis, which is puzzling and this should be a self-correlation. The methodology should be better explained.

*Response: We are sorry that the description of Fig. 3 and methodology of assessment based on DEFs are not described in enough detail. As RC and MCC metrics were calculated based on similar strategies, here we take RC as an example to clarify the details of the method. RC was calculated based on the Pearson correlation coefficient between the FCs for a given pair of sample groups and the corresponding reference values, representing the trend of numerical consistency of FC. The reference FC values were the average of intra-batch FC values from 15 batches, serving as “ground truth” (Additional file 1: Fig. S3). Intra-batch FCs were calculated in each batch which consisted of four sample groups with three replicates per sample group. The primary goal of BECAs is to provide comparable results between cross-batch analysis and within-batch analysis. Hence, intra-batch RC was used as the positive control and calculated as the correlation value between intra-batch FCs with the reference FCs. However, due to lab proficiency and/or technical limitations, variations exist in each batch and lead to differentiation in intra-batch RC values across 15 batches. Hence, intra-batch RC values are not equal to 1. We have revised Fig. 3a, including how to calculate the intra-batch FCs and detect DEFs, and how to compare with reference FCs and DEFs. The description of method for calculating RC of FCs and MCC of DEFs using intra-batch datasets is provided in Lines 791-796 in the revised manuscript. Moreover, the Methods section is carefully edited in the text by highlighting in yellow.*

2. Also, the authors mention that their correlation metrics show better the differences between BECAs than typical performance metrics such as sensitivity and precision, but do not explain why. We know that correlation is a metric that can be biased by extreme values. Is this having an influence in this observation? In any case, an explanation of the difference between evaluation by correlation metrics and the traditional ways of performance evaluation should be provided.

*Response: Thank you for the comments and suggestions. We noticed that this statement was improper and decided to remove it in the revised manuscript. Moreover, according to your following comments of identifying reference DEFs (which is previously known as high-confidence DEFs), we have updated the method of identifying reference DEFs (see response to Question #5 below). Performances across different BECAs have been updated (Fig. 3 and Additional file 1: Fig. S4), with the similar conclusion as before. That is, SVA, RUVs, and ratio-based method exhibited significantly higher performances values in confounded scenario across the three omics types.*

3. The authors state that the ratio-based methods are the best solution for batch correction. I believe that the limitations of this approach should also be highlighted. For example, does the ratio work well regardless the quality of the reference samples? One way to test this is to repeat their analysis taking other samples as reference and evaluating the consistency of the conclusions, or by artificially introducing different noise levels in the references. Moreover, a reference-based method is possible when the introduction of a reference sample can be decided as part of the experimental design. However, this is not always the case of the analysis scenarios that require batch correction. For example, when combining already existing dataset a reference sample may not exist or be possible (i.e. when only two experimental groups are part of the study). Moreover, the method assumes that there is no interaction batch-reference sample. Does this always hold? All these aspects should be discussed, and additional analysis should be included to evaluate the consistency of the reference approach in different data quality scenarios.

*Response: Thank you for the comments and suggestions. The performance of the ratio-based method may be affected by the quality of the reference sample. According to the reviewer’s suggestions, we artificially introduced different levels of noise to the reference samples to evaluate the consistency of ratio-based approach under different data quality scenarios. Specifically, for each feature in each batch, we randomly generated three values of “modified” reference samples using rnorm function in R package with mean zero and SD equal to different folds of SD that were calculated from three replicates of reference samples in the batch. Different folds of SD ranging from 0.1 to 10 were used to mimic different levels of noises in the reference. Ratiobased method was further evaluated using the average of the three “modified” replicates of reference samples as the denominator. SNR across study samples was used to demonstrate efficacy of the ratio-based method.*

*The results showed that the SNR value continuously decreased with deteriorating quality of reference samples, as the reviewer suspected (Additional file 1: Fig. S7). Moreover, with transcriptomics data as an example, when the noise level was increased up to 3-fold SD, the SNR values remained as high as 4.7 in balanced scenario and 10.3 in confounded scenario, which were much higher than original dataset without correction (SNR: -0.3 to -0.9). The results indicated that the ratio-based method performed reasonably well as long as the quality of reference samples was not extremely low. Additionally, SNR values based on noises with 1-fold SD were similar with real datasets, as expected, except in the balanced proteomics dataset. This might be explained by the fact that there were more low-quality datasets in proteomics compared to transcriptomics and metabolomics (Additional file 1: Figs. S1a-c), and lower correlations of replicates of reference samples were detected (Lines 335-351, and Additional file 1: Fig. S1d).*

*Furthermore, according to the comments of the reviewer, we have added a paragraph on the limitations of ratio-based method in the discussion section of the revised manuscript, so that readers can obtain a more comprehensive understanding of this method (Lines 436-464 in the revised manuscript, and see the response to Reviewer 1’s Question #1).*

4. The authors should also bring to the discussion some aspects indicated in the introduction, such as that previous BECAs evaluations provided contradictory results. Why is their study better or how this work sheds lights into previous discrepancies? Where other analysis scenarios considered in the previous studies?

*Response: Thank you for the comments and suggestions. We have added a paragraph in the discussion about comparison between our results with previous BECAs evaluations (Lines 419-435 in the revised manuscript), as follows:*

*“Based on the experimental design composed of specific batches of samples representing balanced or confounded distributions of biological groups, we used standard analysis types such as sample clustering, differential analysis, and predictive modelling to demonstrate comprehensive evaluations of some widely used BECAs.*

*Several findings in this study are consistent, while some are controversial with previous reports. For example, Luo et al. focused on performances of cross-batch prediction in clinical outcomes, and found that ratio-based method outperformed others (Luo J et al., Pharmacogenomics Journal 2010, PMID 20676067). Moreover, Weishaup et al. demonstrated the effectiveness of the RUV method in removing batch effect for molecular subtyping based on various microarray datasets of cerebellar and medulloblastoma, compared to without batch correction, which is consistent with our findings (Weishaupt H et al., Bioinformatics 2019, PMID 30715209). On the other hand, Zhou et al. simulated balanced and confounded datasets and found that BECAs performed well when batch-class was balanced, which is in line with our findings (Zhou L et al., Journal of Genetics and Genomics 2019, PMID 31611172). However, when batch-class was strongly confounded, they found the performances declined in all BECAs under evaluation, including ComBat, SVA and ratio-based method, which appeared to be inconsistent with our findings. This might have been caused by the assumptions underlying the simulated datasets used for evaluation.”*

5. I am puzzled about the definition of high confidence DEF and non-DEF. DEF should be found as such in at least 15% of the batches, while non-DEF should not be found as DEG in more than 50% of the batches. Therefore, a feature that is DEF in 20% of the batches and non-DEF in 80% of the batches will be both high confidence DEF and high confidence non-DEF? This makes no sense to me.

*Response: Thank you for pointing out this. We have changed the strategy for identifying high confidence DEF and non-DEF (now they are named as reference DEF and non-DEF, to be consistent with the accompanying manuscripts from the Quartet project). A list of reference DEFs and non-DEFs were identified based on intra-batch DEFs and nonDEFs using voting for consensus. Specifically, within each batch in each sample pair, a feature could be classified into one of the three groups, i.e. up-regulated DEF, downregulated DEF and non-DEF. The choice with the most first-preference votes from 15 votes from 15 batches was the final classification of the feature. Features receiving more than one first-preference vote were not included in reference datasets. We have provided one supplementary figure to show the step-by-step workflow on the construction of reference datasets (Additional file 1: Fig. S3). Detailed description is also provided in Lines 687-700 in the revised manuscript.*

6. Also, the definition of intra-batch fold-changes could be improved. I assume that the fold-changes are calculated for each feature independently. As there are three replicates for each sample, are the replicates first averaged and then the fold changes computed, or is this done in a different way? Please note that the sentence starting half-way in line 532 is grammatically incorrect.

*Response: Thank you for the comments and correction. Yes, the three replicates were first averaged, and then the fold changes were computed. We have revised the description of intra-batch fold-changes (Lines 674-677) and corrected the grammatical error(s) according to your suggestions. Moreover, we merged the subsections on the description of methods of intra-batch fold-change and intra-batch DEFs into the same subsection as they are highly relevant.*

7. The computation of the relative correlation is also not clear. How are the multiple batches considered here? In the explanatory figure only 3 batches are shown. Do the RC calculations involve only three batches? Since three comparisons are possible, are the values averaged per feature or concatenated to compute correlations?

*Response: Sorry for the confusion due to the lack of clear description of the methods. We have updated the text accordingly (Lines 779-790), as follows:*

*“In each comparison, three replicates of each donor were included, making them suitable for statistical analysis. Specifically, three batches (each batch contained one replicate of a donor) of datasets were randomly selected in balanced scenario, while one batch (each batch contained three replicates of a donor) from each of two donors under comparison was randomly selected in confounded scenario. Cross-batch RC and MCC of DEFs between cross-batch values with reference values were then computed. To eliminate potential selection bias, this process was repeated 15 times. Moreover, since three comparisons were possible, the RC was calculated in each comparison. A total of 45 (15 repeats x 3 sample-pairs) RC and MCC of DEFs values under each BECA method in each scenario and each omics type were obtained. Mean with standard error (s.e.) of RC and MCC of DEFs were further calculated, representing performance of BECAs in identifying differential expression.”*

8. In the calculation of MCC of DEF. Are all batches included in the DEF analysis? Since different number of batches are available for different omics, does this affect the capacity for detection DEF? The authors should reveal how many DEF and non-DEF for each omics type were obtained in the grown-truth and in the evaluation of each BECA method. Readers must understand the numbers of features used to base conclusions.

*Response: Sorry for the confusion. In each omics type, the same number of batches (N=15) are included in the DEF analysis. Numbers of reference DEFs/non-DEFs across three comparisons are: 2,120~2,297/24,589~25,772 for transcriptomics, 55-63/3,421-3,433 for proteomics, and 9-18/52-61 for metabolomics. Detailed numbers in each comparison in each omics type are listed in Additional file 1: Fig. S3.*

9. In the calculation of the ARI, it is unclear what the true clusters are. Are the authors assuming that one cluster corresponds to the multiomics samples of the same group? If so, it is to be expected that the same within-group similarity is maintained across omics layers? This is not obvious to me, and the assumptions of this analysis should be clearly stated and discussed.

*Response: Yes, we assume that one cluster corresponds to samples of the same group and set them as the true clusters, because the Quartet multiomics materials, including RNA, protein and metabolite, are derived from the same batch of cultured cells. Moreover, RNA, protein and metabolite reference materials were established in the same batch. Hence, it is expected that the same within-group similarity is maintained across omics layers. Replicates from the same sample group should clustered together, regardless of within-omics or cross-omics. We have added information of true clusters in the revised manuscript (Lines 293-299).*

10. Please include some extra information about the multiomics datasets. Number of features in each case and treatment of missing values. Although this might be described in the respective papers, the information should be included in this paper for clarity.

*Response: Thank you very much for the suggestions. We updated the Methods section with extra information about the multiomics datasets in the revised manuscript. Specifically, descriptions of treatment of missing values of proteomics and metabolomics data are provided in Lines 559-568 and Lines 586-590, respectively. There are no missing values in transcriptomics data. The number of original features is 58,395, 8,150, and 984 for transcriptomics, proteomics, and metabolomics, respectively. After filtering, the number of features considered to be detected across multiple batches and used in further analysis in each omics type were 26,907, 3,489, and 71 for transcriptomics, proteomics, and metabolomics, respectively. Detailed information is included in the revised manuscript in Lines 659-669 and Additional file 1: Fig. S3.*

**Second round of review**

**Reviewer 1**

In the revised version of their manuscript, the authors have addressed all points raised by the reviewers. The additional clarifications make this version better to read and understand. I would like still to mention the following minor comments:

* Injecting random statistical noise in the data do not simulate lower biological sample quality. I would define it more like a simulation of a lower measurement precision.

* Figure 1B. One can read "4 groups x 3 reps per group" but one can see Batch 1' below with 3 groups of 4 colored boxes. The total is still 12 but shouldn't it fit the text description?

**Authors Response**

**Point-by-point responses to the reviewers’ comments:**

In the revised version of their manuscript, the authors have addressed all points raised by the reviewers. The additional clarifications make this version better to read and understand. I would like still to mention the following minor comments:

*Response: We greatly appreciate the reviewer’s positive assessment of our revision. The reviewer’s constructive comments and suggestions are very helpful for us to further improve our manuscript.*

* Injecting random statistical noise in the data do not simulate lower biological sample quality. I would define it more like a simulation of a lower measurement precision.

*Response: Thank you very much for your important comment. We agree with the reviewer that injecting random statistical noise is more like a simulation of a lower measurement precision. Here, we conducted additional simulation analysis in transcriptomics data to better simulate reference samples with low-quality, and found that the ratio-based method performed reasonably well as long as the quality of reference samples was not extremely low.*

*In the new simulation analysis, we artificially reduced the read counts of some genes to simulate reference samples with low-quality. A low-quality sample is usually characterized with RNA degradation and such degradation is usually gene specific, that is, some genes are degraded more severely while others do not. The fragmentation of RNA molecules in the low-quality sample can result in a decrease in the complexity of the RNA library, leading to some RNA fragments not detectable and therefore lower count values in the RNAseq analysis (PMID: 23685885, 24885439). Here, for each replicate of the reference sample, we randomly selected a certain percentage of genes and artificially reduced their read counts to 1/2, 1/4, or 1/8 of the original level (Additional file 1: Fig. S8 in the revised manuscript). Using this simulation strategy, the affected genes across different replicates in the same batch were probably different, which was as expected. Modified read counts in each sample were then normalized to Count Per Million (CPM). Ratio-based profiles were further conducted based on log2-transformed CPM using the average of the three replicates of reference samples as the denominator. SNR across study samples was used to demonstrate the effectiveness of the ratio-based method.*

*The results showed that the SNR value continuously decreased with deteriorating quality of reference samples, as the reviewer suspected (Additional file 1: Fig. S8 in the revised manuscript). However, it should be noticed that when up to 10% of detected genes were reduced to 1/8 of their original counts, the SNR values remained as high as 17.1 and 14.8 in balanced and confounded scenario, respectively, which were much higher than the original dataset without correction (SNR: -0.3 to -0.9). Moreover, even when the percentage of affected genes were increased up to 30%, the SNR values in confounded scenario remained 14.5. The results indicated that as long as the quality of reference samples was not too low, the ratio-based method remained effective.*

* Figure 1B. One can read "4 groups x 3 reps per group" but one can see Batch 1' below with 3 groups of 4 colored boxes. The total is still 12 but shouldn't it fit the text description?

*Response: Thank you very much for your comments. We are sorry for the confusion from reading Figure 1B. According to the reviewer’s suggestion, we have revised Figure 1B to "4 groups x 3 reps per group" in the revised manuscript*
